# Waterpipe smoke and e-cigarette vapor differentially affect circadian molecular clock gene expression in mouse lungs

**DOI:** 10.1371/journal.pone.0211645

**Published:** 2019-02-27

**Authors:** Naushad Ahmad Khan, Shaiesh Yogeswaran, Qixin Wang, Thivanka Muthumalage, Isaac K. Sundar, Irfan Rahman

**Affiliations:** Department of Environmental Medicine, University of Rochester Medical Center, Rochester, New York, United States of America; University of Texas Medical Branch at Galveston, UNITED STATES

## Abstract

The use of emerging tobacco products, such as waterpipe or hookah and electronic cigarettes (e-cigs), has gained significant popularity and are promoted as safer alternatives to conventional cigarettes. Circadian systems are internal biological oscillations that are considered important regulators of immune functions in mammals. Tobacco induced inflammatory lung diseases frequently exhibit time-of-day/night variation in lung function and symptom severity. We investigated the impact of inhaled e-cig vapor and waterpipe smoke (WPS) on pulmonary circadian molecular clock disruption by determining the changes in expression levels and abundance of core clock component genes (BMAL1, CLOCK) and clock-controlled output genes (*Rev-erbα*, *Per2*, *Rev-erbβ*, *Cry2*, *Rorα*) in mouse lungs. We showed that the expression levels of these pulmonary core clock genes and clock-controlled output genes were altered significantly following exposure to WPS (*Bmal1*, *Clock*, *and Rev-erbα)*. We further showed a significant yet differential effect on expression levels of core clock and clock-controlled genes (*Bmal1*, *Per2)* in the lungs of mice exposed to e-cig vapor containing nicotine. Thus, acute exposure to WPS and e-cig vapor containing nicotine contributes to altered expression of circadian molecular clock genes in mouse lungs, which may have repercussions on lung cellular and biological functions.

## Introduction

Within the past decade alternative tobacco products have gained popularity. There has been a surge in the usage of non-cigarette based tobacco products, such as waterpipe smoke (WPS) and electronic cigarettes (e-cigs), particularly among adolescents, young adults, and those trying to quit traditional tobacco-based cigarette smoking. Waterpipe, often referred to as hookah, narghile, and shisha, is a traditional method of smoking tobacco which involves passage of charcoal heated air through a perforated aluminum foil to generate smoke which bubbles through water before being inhaled [1]. E-cigs are electronic devices used for inhaling vapor containing nicotine without tobacco; these devices were originally developed to aid smoking cessation as well as a low-risk alternative to traditional tobacco-based cigarettes [2]. A growing body of scientific evidence suggests that WPS and e-cig vapor have implications on pulmonary pathophysiology and lung injurious responses [3]. Chronic obstructive pulmonary disease (COPD) is characterized by irreversible airflow limitation and abnormal inflammatory responses in the lung. It has been shown that WPS can lead to a decline in forced expiratory volume in 1s (FEV_1_) and peak expiratory flow rate (PEFR), indicating a possible role in the development of COPD [4]. Similarly, chronic exposure to e-cig vapors containing nicotine has been shown to induce features of COPD in mice and human airway cells, which suggests inhalation of e-cig vapor can manifest as airway and lung diseases [5]. However, the effects of WPS and e-cig vapor containing nicotine on different lung pathophysiological events are not known.

Circadian rhythms represent intrinsic biological oscillations that synchronize various cellular and physiological functions in mammals throughout a 24 h period driven by the autonomous circadian system [6]. This clock regulates the daily light/dark cycle which is associated with the sleep-wake cycle, core body temperature, hunger, as well as other physiological processes [7]. In mammals, the central clock is localized in the suprachiasmatic nuclei (SCN), located in the basal part of the hypothalamus. The peripheral clock in the lung is managed by core clock and organ-specific clock-controlled output genes (CCGs) [8]. The physiological processes which occur in all other organs, including the lungs, are governed by these core clock proteins [9]. Core clock proteins are a part of a continuous auto-regulatory feedback loop within each cell of the lungs; consequently, any disruption of this feedback loop can lead to lung pathophysiology. The physiological processes occurring within the lung are governed by timing mechanisms regulated by a transcription/translational based feedback oscillator. This feedback oscillator is made up of critical molecular clock proteins like brain and muscle ARNT-like 1 (BMAL1), circadian locomotor output cycles protein kaput (CLOCK), period circadian regulator 2 (PER2), and nuclear receptor subfamily 1 group D member 1 (NR1D1 or REV-ERBα) [10].

To date, there are limited studies that have examined the effect of clock dysfunction in lung pathophysiology. Recently, we and others have provided evidence which suggests that cigarette smoke (CS) has a profound role in disrupting pulmonary circadian clock rhythmicity particularly in airway cells that can impede both pulmonary circadian rhythm and lung function, augment oxidative stress, inflammation, and lead to cellular senescence and DNA damage [10–13]. Lung clock alteration by environmental agents/tobacco smoke can have repercussions in the pathophysiology of COPD and its exacerbations [14, 15]. While most of the studies have investigated the effect of CS exposure induced pulmonary circadian clock disruption [10, 11, 13, 16, 17], the effects of alternative non-cigarette tobacco products, such as WPS and e-cig vapor containing nicotine on pulmonary circadian molecular clock protein abundance and gene expression are not known. This lack of knowledge necessitates the use of animal models in acute and chronic exposures to elucidate the role of WPS and e-cigs in exacerbating pulmonary circadian molecular clock disruption.

We tested the hypothesis that WPS and e-cig vapor containing nicotine can perturb the expression of molecular clock proteins/genes in the lung that can alter the cellular and molecular functions during the pathogenesis of airway diseases. To determine the effect of acute exposure to WPS and e-cig vapor containing nicotine, we analyzed the changes in protein abundance and gene expression levels of the circadian molecular clock genes in mouse lungs.

## Materials and methods

### Ethics statements: Animal study protocol and institutional biosafety approvals

All animal protocols and procedures described in this study were approved by the University Committee on Animal Research of the University of Rochester, Rochester, NY. All experiments performed in this study were approved and in accordance with the University of Rochester Institutional Biosafety Committee.

### Experimental animals

Adult C57BL/6J mice of both sexes were purchased from the Jackson Laboratory (Bar Harbor, ME) (body weight 25–30 gms; 14–16 weeks old for WPS exposure); (body weight 22–32 gms; 16–20 weeks old for e-cig nicotine exposure) and were housed in an inhalation facility under controlled conditions of temperature, humidity, and a pathogen-free environment at the University of Rochester for one week acclimatization before WPS and nicotine exposures. All the mice were maintained under a 12:12 light:dark cycle (lights on between 6 AM to 6 PM). The animals were kept in cages and were fed a regular diet (pelleted food) and water *ad libitum*, unless otherwise indicated. The animals were monitored on a daily basis during exposure to check for any signs of distress. The mice were anesthetized 24 h after the last WPS exposure and two hours after the last e-cig exposure by injecting a single dose of (90 mg/kg body weight) pentobarbital sodium (Abbott Laboratories, Abbott Park, IL, USA) and were euthanized by exsanguination. Lung tissues from mice exposed to ten days of acute WPS and three days of acute nicotine-containing e-cigarettes vapors were used in this study.

### Whole-body waterpipe smoke exposure

We used a whole-body exposure model for exposing the animals to waterpipe smoke. The waterpipe exposure system consisted of a waterpipe smoking machine set up and an exposure chamber. This exposure system has been used by us in a recent study [1]. Smoke generated by charcoal heated tobacco-molasses was bubbled through water using an FMI pump at one-liter per minute. The exposure was designed to simulate the human puff topography by producing three puffs per minute with each puff lasting for three seconds and a seventeen-second inter-puff interval by modifying the Beirut method [1]. By interconnecting two of these standalone hookah pipes (Mya and Guru brands) to a HEPA filtered room air supply the final concentration of WPS was diluted approximately 4.3 times in the exposure chamber. For one hour of WPS exposure approximately twelve grams of flavored tobacco (mixed fruit, plum, nectarine, and cocktail) were used in each hookah exposure session. Total particulate matter (TPM) was determined gravimetrically through a sampling port and the CO levels were monitored with an E1 USB CO data logger (Lascar Electronics, NH, USA) placed inside the exposure chamber to assess the WPS exposure conditions.

Four-month-old C57BL/6J mice were subjected to whole-body WPS exposure by placing them in a metal wire cage inside the WPS exposure chamber. Their smoke unexposed counterparts were exposed to HEPA filtered room air in an identical cage setup. All the mice were exposed to WPS during day time between 10 am to 2 pm for 10 consecutive days. Eight mice per group were exposed to either WPS or filtered room air (control) for ten consecutive days: thirty-minute exposures twice a day with a one-hour interval for the first three days and one-hour exposures twice a day with a one-hour inter-exposure interval for the remaining 7 days. Subsequently, the mice were euthanized twenty-four hours post exposure and the tissues were collected and stored for the designated assays.

### E-cigarettes and e-liquids with nicotine

The electronic nicotine delivery systems (ENDS) device used for the exposure was Joytech eVIC VTC mini (SciReq, Montreal, QC, Canada), which is a rechargeable, fixed, automatic device purchased from SciReq. The e-liquids were poured into a standard tank and then attached to the e-cig device. All e-cig components were purchased from SciReq and the atomizer/coil (0.15 Ω) was purchased online from Kanger Tech (Shenzhen, China). The atomizer was replaced after each exposure every day to avoid overheating of the coil. The e-liquids were purchased online from xtremevaping.com. E-liquid containing 100% propylene glycol (PG) alone and an unflavored base consisting of 100% PG with 25 mg/mL nicotine (Lot#57724) were used in this study.

### E-cigarette nicotine exposure

After a week of acclimatization, the *in vivo* mouse e-cig exposure was performed inside a fume hood using a whole body SCIREQ “InExpose” smoking system (SCIREQ Inc., Montreal, Canada). The system consisted of a stroke controller that activates the rechargeable e-cig device (Joytech eVIC VTC mini, SciReq) filled with e-liquid. This custom-built stroke controller was regulated by the Scireq flexiware software (Version 8.0) that automatically controls the stroke duration and delivers the input to modulate puff profile based on realistic topography data from e-cig users. The software also controls the exposure temperature, humidity, oxygen, and carbon dioxide, as well as the exposure time inside the whole-body exposure chamber [18]. The e-cig exposure was set to a two hour protocol that includes a puff profile of 2 puffs/min (70 mL puff volume). The duration of each puff was 2–3 sec long with a 30 sec inter-puff interval (dilution air) between the puffs. The e-cig vapors were drawn through the pump 1 of the e-cig device and diluted with the room air into the buffer chamber before being drawn into the whole-body chamber. The flow rate of the pump that draws the e-cig aerosol was set at 1.0 L/min with a constant flow of dilution air (room air) into the chamber. The flow rate of the pump 2 that constantly draws air from the whole-body chamber was set at 2 L/min for the duration of the profile. Mice were exposed to inhaled e-cig vapors containing PG with nicotine or PG alone for three consecutive days. Mice were randomly divided into air, PG alone, and PG with nicotine groups and were exposed to e-cig vapors 2 hr/day for 3 days. All the mice were exposed to e-cig containing nicotine or PG alone during the daytime (between 10 am to 1 pm) for 3 consecutive days. Air group (room air exposed) mice were placed in the same room to ensure similar conditions such as temperature, noise, and light intensity during acute e-cig exposure.

### Protein extraction from lung tissue

Following sacrifice, lung tissues were blotted dry on a filter paper and stored at -80°C until further use. Lung tissue was mechanically homogenized with 0.4 ml of radioimmunoprecipitation assay buffer (RIPA) containing protease inhibitor cocktail (Millipore Sigma) and the lung tissue homogenates were kept on ice for 45 min to allow complete cell lysis. This step was followed by centrifugation of lysates at 12,000 *g* for 10 minutes. The supernatant was subsequently collected as lung homogenate, then aliquoted and stored at -80°C for western blot analysis. The total protein concentration of the supernatant was measured with a bicinchoninic acid (BCA) kit (Pierce, Rockford, IL) using bovine serum albumin as the protein standard.

### Immunoblot analysis

Protein samples (20 μg) from lung homogenates of the mice exposed to waterpipe smoke or e-cig vapors were separated by 10% SDS-PAGE gel. Separated proteins were electroblotted onto nitrocellulose membranes (Amersham, Arlington Heights, IL, USA). The membranes were blocked for 1 h at room temperature with 5% non-fat milk to block nonspecific binding sites. Membranes were then probed with specific primary antibodies anti-BMAL1 (Abcam ab93806, dilution ratio 1:2000), anti-REV-ERBα (Cell signaling 13418S, dilution ratio 1:1000), anti-PER2 (Invitrogen, PA5-23339, dilution ratio: 1:500), and anti-CLOCK (Santa Cruz, sc-25361, dilution ratio: 1:200), which were then incubated overnight at 4°C to determine the presence of respective proteins. The membranes were subjected to four 10-minute washes and the membranes were probed with suitable secondary anti-rabbit antibodies (1:10,000 dilution in 5% non- fat milk) linked to horseradish peroxidase for 1 h. The bound complexes were then detected using the enhanced chemiluminescence method (Perkin Elmer, Waltham, MA) following the manufacturer’s protocol. The images were taken with Bio-Rad ChemiDoc MP imaging system. The blots were subsequently stripped and equal loading of the samples was determined by re-probing membranes with β-actin (Abcam ab20272, dilution ratio, 1:2000). Band intensity was determined by densitometry analysis and expressed as fold change relative to corresponding loading control.

### Enzyme-Linked Immunosorbent Assay (ELISA) of circadian clock proteins

The levels of BMAL1, NR1D1, and PER 2 were measured in undiluted lung homogenates of mice exposed to WPS (10-day exposure) and e-cig vapors containing nicotine (3-day exposure) using pre-coated quantitative sandwich ELISA kits according to the instructions from the manufacturer (BMAL-1, Catalog # MBS9328769; NRID1, catalog # MBS9340203; and PER-2, Catalog # MBS9909846, MyBioSource Inc, San Diego, CA, USA). We used the same air group control samples for both waterpipe and e-cig ELISA assays. Both standards and samples were assayed in duplicates, and levels of clock proteins were determined by measuring OD at 450 nm using a microplate reader (Bio-Rad, San Diego, CA).

### Isolation of total cellular RNA and mRNA analysis

The lungs of the mice exposed to WPS or e-cig vapors containing nicotine or PG alone were snap frozen and stored at -80°C. One hundred milligrams of frozen lung tissues were homogenized in Trizol and total RNA was isolated using the RNeasy kit (Qiagen), according to the manufacturer’s instructions. The extracted RNA was quantitated by absorbance using a nanodrop spectrophotometer (Nanodrop One; Thermo Scientific) and was followed immediately by cDNA synthesis using the First Strand cDNA synthesis kit (Qiagen). One μg of total RNA was used for the initial reaction. cDNA was stored at -20°C and was used for quantitative real-time polymerase chain reaction (qPCR). For normalization 18S ribosomal RNA was used as a control. The CT value for 18S ribosomal RNA was subtracted from the CT value for the gene of interest to obtain a delta CT (ΔCT) value. The relative fold-change for each gene was calculated using the 2^-ΔΔCt^ method. The mouse circadian clock genes primer pairs used are listed in Table 1.

**Table 1:** Primer Sequences for mouse circadian clock genes.

Gene Name	Primer Sequences
*Bmal1*	Forward: AAGGGCCACTGTAGTTGCTG Reverse: CTGCAGTGAATGCTTTTGGA
*Clock*	Forward: GGAGTCTCCAACACCCACAG Reverse: GGCACGTGAAAGAAAAGCAC
*Nr1d1 (Rev-erbα)*	Forward: GAGTCAGGGACTGGAAGCTG Reverse: AAGACATGACGACCCTGGAC
*Nr1d2 (Rev-erb β)*	Forward: TGGAGGCAGAGCTAGAGGAA Reverse: ACCCGGTGCTCATGATGT
*Cry2*	Forward: TCCCCGGACTACAAACAGAC Reverse: GTCTACATCCTCGACCCGTG
*Rorα*	Forward: TTGCAGCCTTCACACGTAAT Reverse: AGGCAGAGCTATGCGAGC
*Per2*	Forward: CTTGGGGAGAAGTCCACGTA Reverse: TACTGGGACTAGCGGCTCC

### Statistical analysis

The statistical analysis of significance was calculated using the unpaired Student’s *t*-test or one-way ANOVA followed by Tukey’s *posthoc* test for multiple comparisons by GraphPad Prism Software version 7.0 (La Jolla, CA). We also performed Grubb’s test for detecting outliers to make sure all the data points were accounted for data analyses. The results are shown as the mean ± SEM. *P* < 0.05 was considered statistically significant.

## Results

### Waterpipe smoke and e-cig vapor containing nicotine differentially affected the abundance of circadian clock proteins in lung tissue

**Fig 1:**
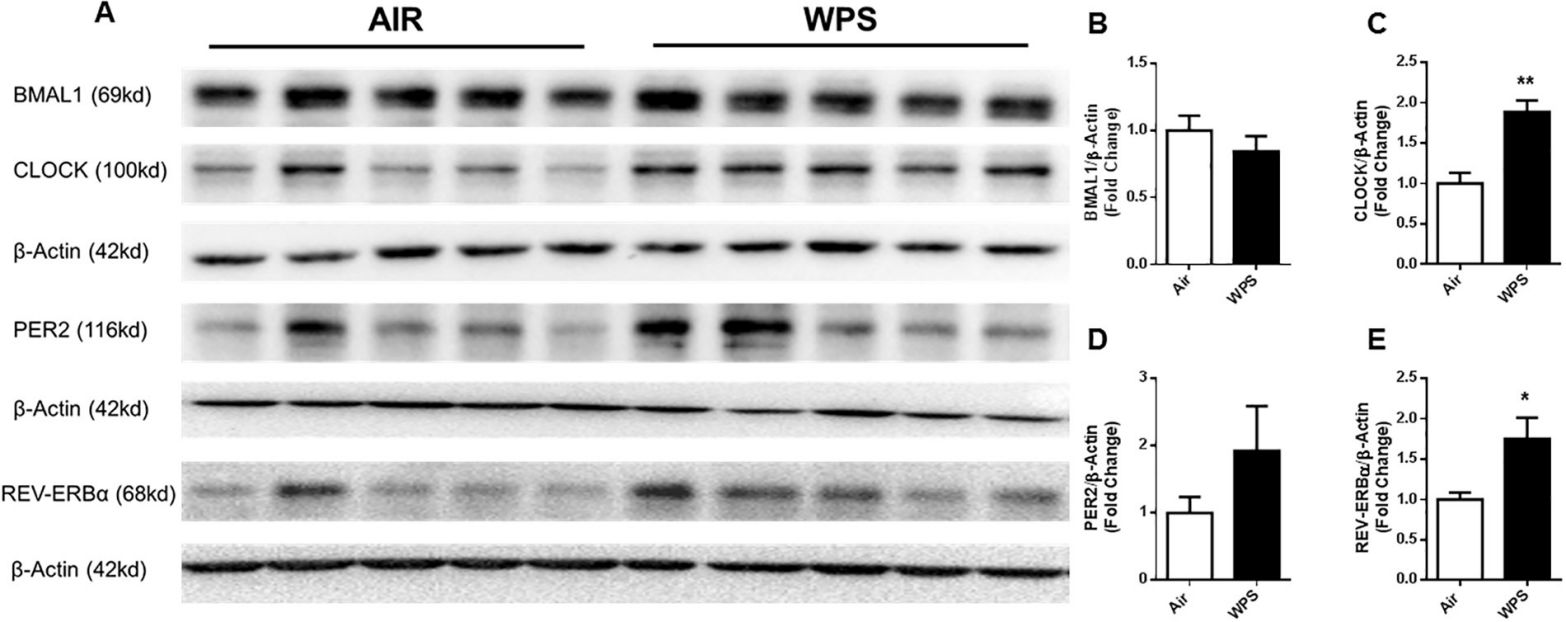
Acute waterpipe smoke (WPS) exposure altered the levels of circadian molecular clock proteins in mouse lung. C57BL/6J mice were exposed to air or WPS for 10 days and euthanized 24-hrs post-last exposure. Abundance of clock and clock-controlled proteins were analyzed in whole lung homogenate samples by immunoblot analysis. β-Actin bands shown for BMAL-1 and CLOCK are identical as both proteins were probed on the same membrane. The band intensity was measured by densitometry analysis and data were presented as fold change relative to β-actin loading control. Ten out of the twelve samples from the full gel/blot were included in the figure (S1 Fig). Full gels/blots with bands (unedited/uncropped electrophoretic gels/blots) derived from the same experiments were shown (S1 Fig). Data are shown as mean ± SEM, n = 5 per group. Statistical significance was calculated using the unpaired Student’s *t*-test *P < 0.05, **P < 0.01 vs. air controls.

We measured clock protein levels, such as BMAL1, CLOCK, PER2, and REV-ERBα, in mouse lung homogenates to determine WPS and e-cig induced alterations in circadian molecular clock proteins. Acute 10 day WPS exposure reduced BMAL1 protein abundance compared with air-exposed controls (Fig 1A and 1B). Surprisingly, we found acute WPS exposure significantly increased CLOCK (*P*< 0.01) and PER2 (though not significant) protein levels compared with air-exposed controls (Fig 1A, 1C and 1D). Additionally, we found REV-ERBα protein levels were increased significantly following WPS exposure compared with air-exposed control (*P <* 0.05) (Fig 1A–1E).

**Fig 2:**
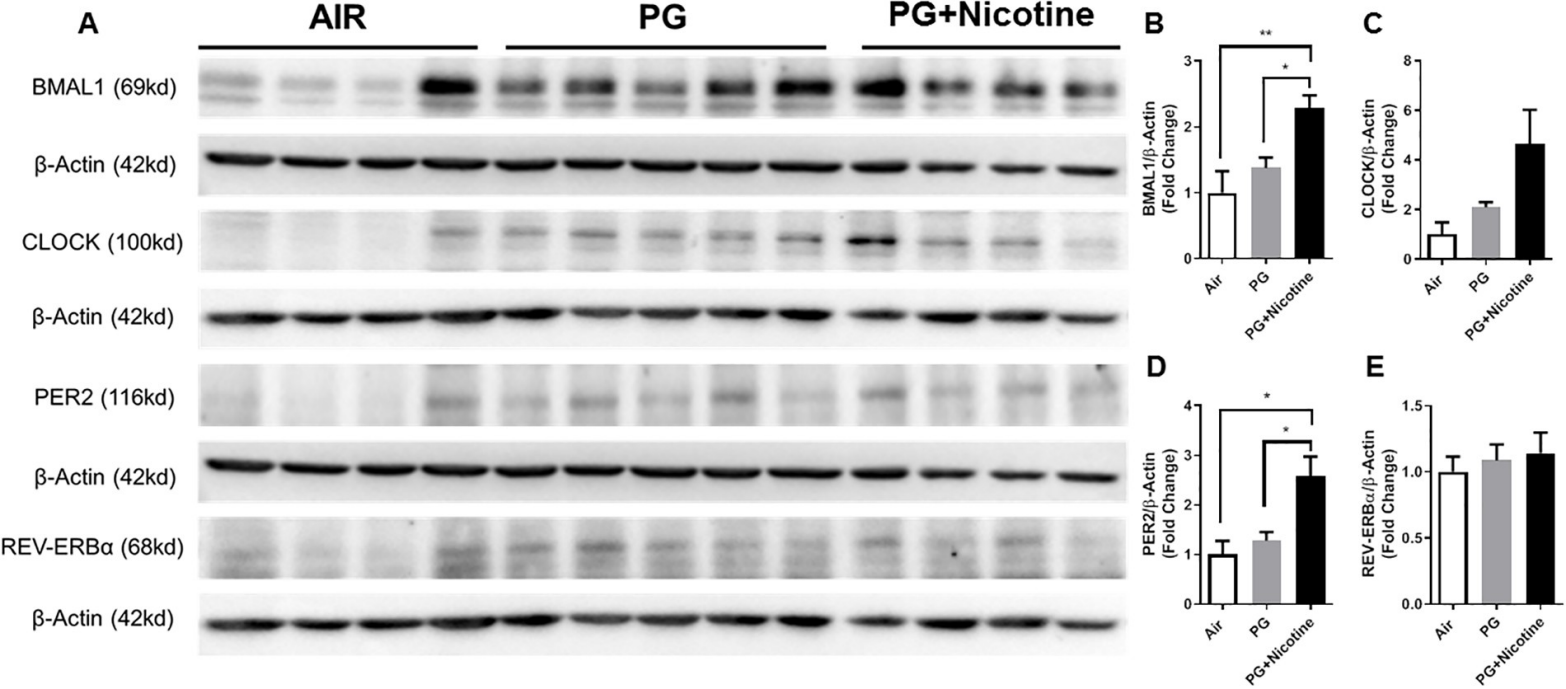
Acute exposure to inhaled e-cig vapor containing nicotine showed altered levels of circadian molecular clock proteins in mouse lung. C57BL/6J mice were exposed to e-cig vapor containing nicotine (25mg/ml) or propylene glycol (PG) alone for three days (2 hrs/day) and euthanized 2-hrs post-last exposure on day 3. Abundance of circadian clock proteins was measured in lung homogenates by immunoblot analysis. β-Actin bands shown for BMAL-1 and PER-2 are identical as both proteins were probed on the same membrane. Similarly, β-Actin bands shown for CLOCK and REV-ERB*α* are identical as both proteins were also probed on the same membrane. The band intensity was measured by densitometry analysis and data were presented as fold change relative to β-actin loading control. Full gels/blots with bands (unedited/uncropped electrophoretic gels/blots) derived from the same experiments were shown (S2 Fig). Data are shown as mean ± SEM, n = 4 for air and PG+Nicotine group and n = 5 for PG group. Only four of the five samples in the PG+Nicotine group from each full gel/blot targeted for each protein were included in the figure (S2 Fig). Statistical significance was determined by one-way ANOVA followed by Tukey’s post hoc test for multiple comparisons. *P < 0.05, **P < 0.01 vs. air or PG.

Acute 3-day e-cig exposure significantly increased BMAL1 and PER2 protein abundance in PG with nicotine group compared with air controls and PG alone groups (Fig 2A, 2B and 2D). We found CLOCK protein levels were modestly increased in PG and nicotine exposed mice compared with air controls and PG alone groups (Fig 2A and 2C). Furthermore, REV-ERBα levels remained unaffected by PG alone and PG with nicotine exposed mice compared to air-exposed controls (Fig 2E). Based on our results, we found acute exposure to WPS and e-cig vapor containing nicotine differentially affects clock proteins, such as BMAL1, CLOCK, PER2, and REV-ERBα levels in the lungs.

### Circadian clock protein abundance is differentially altered by acute exposure to waterpipe smoke and e-cig vapor containing nicotine in mouse lung

**Fig 3:**
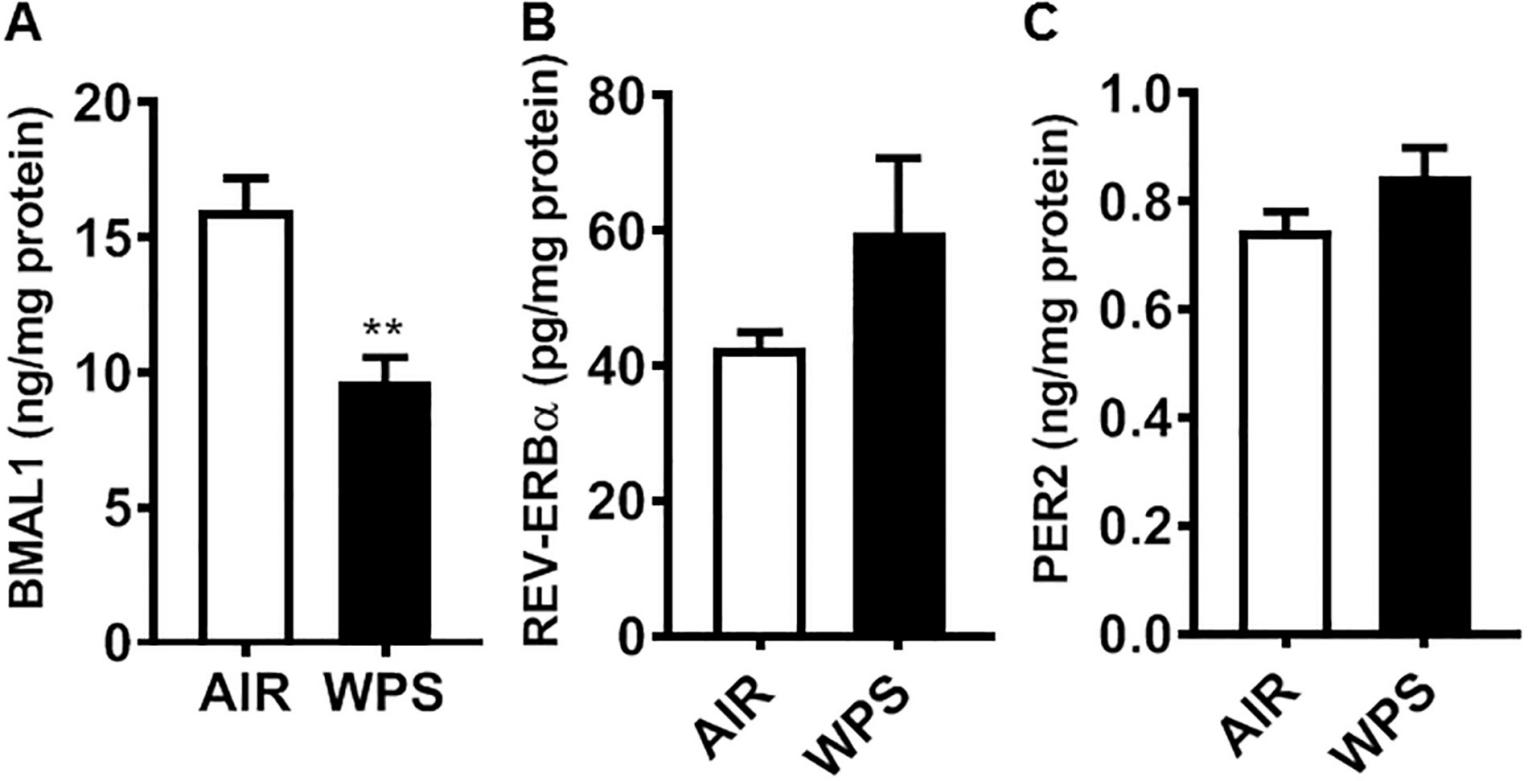
ELISA analysis showed altered levels of circadian molecular clock proteins in mice exposed to acute WPS. C57BL/6J mice were exposed to air or WPS for 10 days and euthanized 24 hrs post-last exposure. Levels of BMAL1, NR1D1 (REV-ERB*α*), and PER2 were measured in lung homogenates by ELISA as per manufacturer’s instructions. Data are shown as mean ± SEM, n = 4–7 per group except for BMAL1 ELISA we used n = 84 for air-exposed mice. Statistical significance was calculated using the unpaired Student’s *t*-test. **P < 0.01 vs. air controls.

In order to complement the changes that we observed by immunoblot analyses we performed ELISA using the lung homogenate samples from WPS and e-cig vapor exposed mice. BMAL1 protein levels were significantly reduced in mice exposed to WPS compared to air-exposed controls (*P* < 0.01) (Fig 3A). WPS exposed mice showed a modest increase in REV-ERBα and PER2 protein levels (though not significant) compared to air-exposed controls (Fig 3B and 3C).

**Fig 4:**
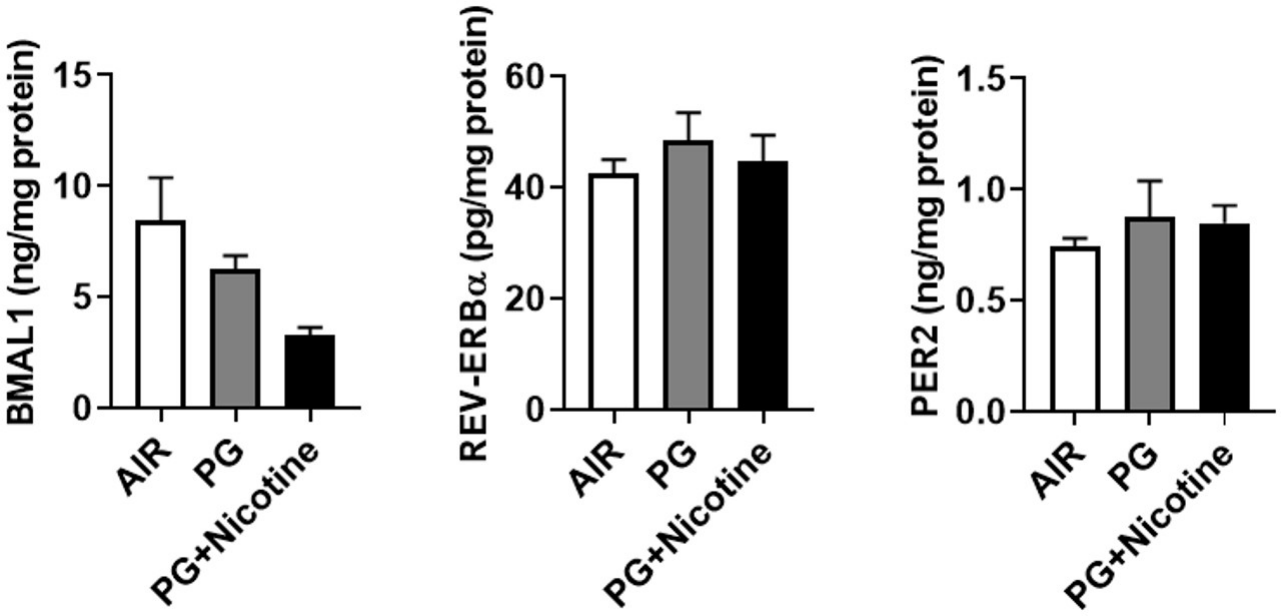
ELISA analysis showed altered levels of circadian molecular clock proteins in mice exposed to inhaled e-cig vapor containing nicotine. C57BL/6J mice were exposed to e-cig vapor containing nicotine (25 mg/ml), propylene glycol (PG) alone and air as a control (2 hrs/day) and euthanized 2 hrs post-last exposure on day 3. The levels of BMAL1, NR1D1 (REV-ERB*α*), and PER2, were measured in lung homogenates by ELISA as per manufacturer’s instructions. Data are shown as mean ± SEM, n = 5–6 per group.

Following acute exposure to e-cig vapor, the levels of BMAL1 protein showed an insignificant change in PG with nicotine and PG alone groups compared to air-exposed controls (Fig 4A). REV-ERBα protein levels did not show any change in mice exposed to PG with nicotine compared with air control and PG alone groups (Fig 4B). PG with nicotine exposed mice showed increased PER2 compared with air control and PG alone groups (Fig 4C). Overall, our ELISA data results corroborated the immunoblot analysis shown earlier.

### Waterpipe smoke and e-cig vapor differentially altered the expression of circadian clock genes in mouse lung

**Fig 5:**
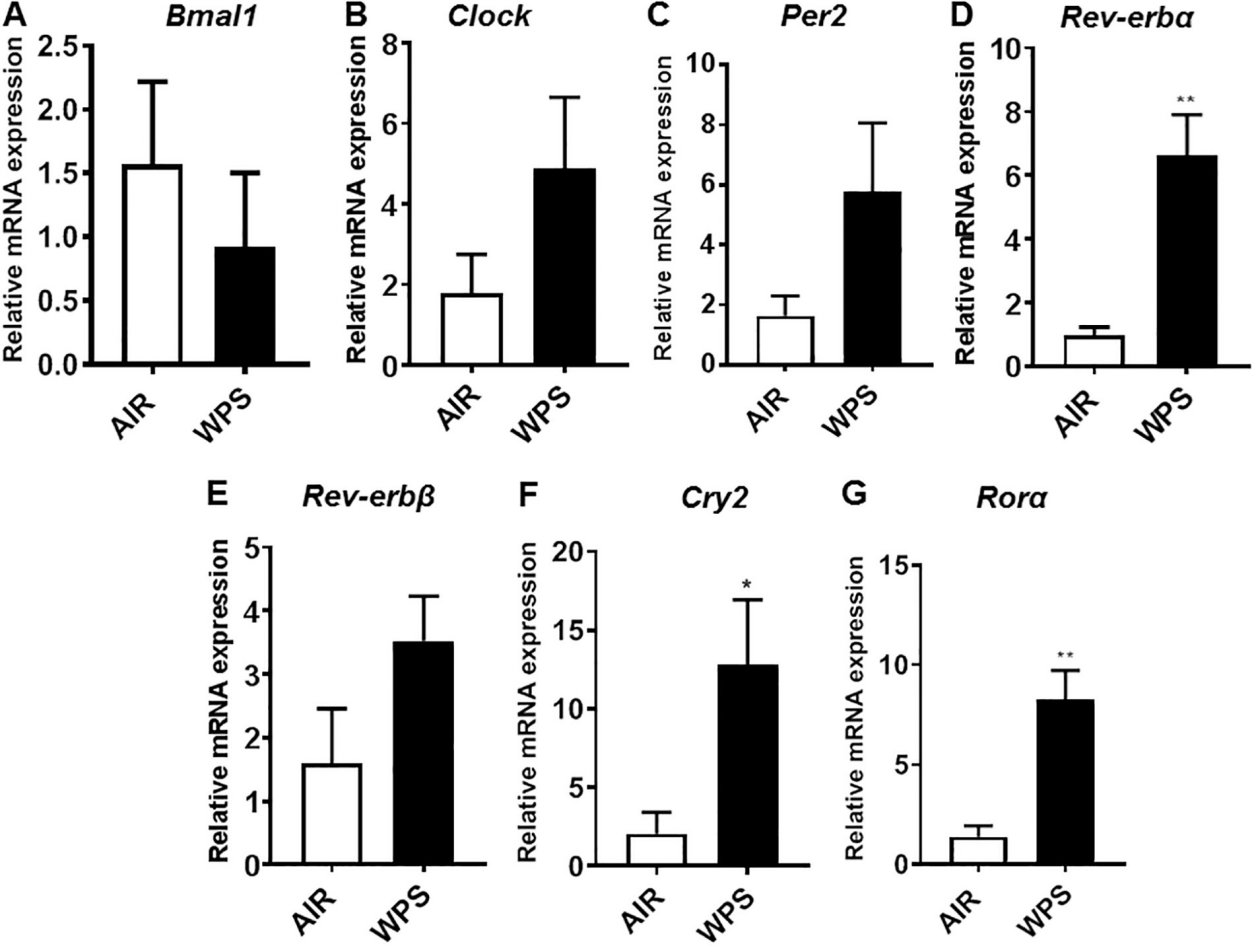
Acute exposure to waterpipe smoke exhibited changes in the expression of circadian molecular clock genes. C57BL/6J mice were exposed to air or WPS for 10 days and euthanized 24-hrs post-last exposure. mRNA expression of circadian clock genes was measured using qPCR analysis from RNA extracted from the lung tissues of mice exposed to WPS and air. Data are shown as mean ± SEM, n = 6–7 per group. Statistical significance was calculated using the unpaired Student’s *t*-test. *P < 0.05, **P < 0.01, vs. air controls.

Expression of clock-controlled genes in the lungs of mice exposed to acute WPS and e-cig vapor containing nicotine was performed using quantitative PCR. Consistent with our immunoblot analysis for mice exposed to WPS, we observed a reduction in the expression of *Bmal1* compared with air controls (Fig 5A). There was a modest increase in the mRNA expression of *Clock* and *Per2* in WPS exposed mice compared with air controls (Fig 5B and 5C). Further, we found a significant increase in mRNA expression of *Rev-erbα* (*P* < 0.01) and a modest increase in *Rev-erbβ* in mice exposed to WPS compared with air-exposed controls (Fig 5D and 5E). More specifically, acute exposure to WPS caused a significant increase in the expression of *Cry2* (*P* < 0.05) and *Rorα* genes (*P* < 0.01) in lungs of mice exposed to WPS compared with air-exposed controls (Fig 5G–5F).

**Fig 6:**
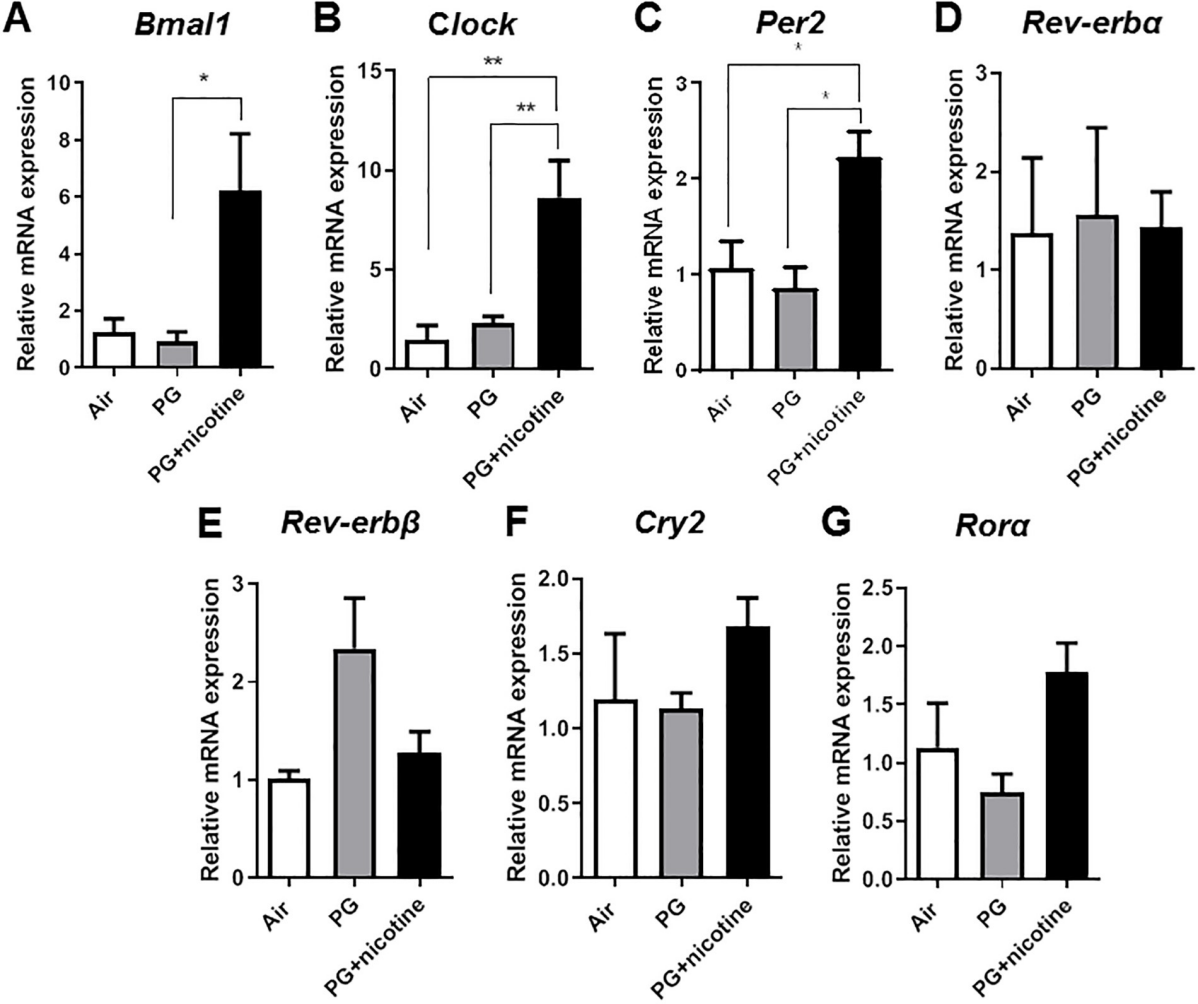
Acute exposure to e-cig vapor containing nicotine alters the expression of circadian molecular clock genes. C57BL/6J mice were exposed to e-cig vapor containing nicotine (25 mg/ml) or propylene glycol (PG) alone for 3 days (2 hrs/day) and euthanized 2 hrs post-last exposure on day 3. mRNA expression of clock and clock-controlled genes were measured using qPCR analysis from the lung tissues of mice exposed to PG with nicotine, PG alone, and air as a control. Data are shown as mean ± SEM, n = 5 per group. Statistical significance was determined by one-way ANOVA followed by Tukey’s multiple comparison test. *P < 0.05, **P < 0.01 vs. PG alone or air controls.

Further, in corroboration with our immunoblot analysis, acute 3-day e-cig exposure (PG with nicotine) significantly increased the expression of *Bmal1* in the lungs compared with air control and PG alone groups (*P<*0.05) (Fig 6A). Additionally, we also observed a significant increase in the expression of *Clock* and *Per2* genes in mice exposed to PG with nicotine compared with air control and PG alone groups. (Fig 6B and 6C). The expression of clock associated nuclear receptor genes, *Rev-erbα* and *Rev-erbβ*, was relatively unaltered in mice exposed to PG with nicotine compared with air control and PG alone groups (Fig 6D and 6E). We did not detect gene transcript changes for either *Rev-erbα* or *Rev*-*erbβ*; nonetheless there was a marked upregulation of *Bmal*1 mRNA suggesting a loss of REV-ERB negative feedback. Additionally, acute exposure to e-cig vapor containing nicotine also caused a modest increase in the expression of *Cry2* and *Rorα* genes in mouse lungs (Fig 6F and 6G).

Our data suggest that the mechanism of regulation of molecular clock genes due to exposure to non-cigarette tobacco products occurs at the level of transcription.

Further, as evident from our immunoblot and qPCR data analysis, WPS and e-cig vapors containing nicotine differentially alter the expression of these genes in mouse lung. Lastly, our protein abundance results corroborate with the gene expression data.

## Discussion

The central pacemaker is located within the SCN in the hypothalamus [19]. Peripheral tissues like lung are also able to oscillate independently and are synchronized with external environmental stimuli by the central pacemaker in the SCN [7, 8, 20]. Any disruption in the timing or amplitude of circadian clock genes at the tissue level can lead to circadian misalignment between central and peripheral oscillators which can result in abnormal respiratory functions, inflammatory responses, and mucus hypersecretion. Environmental smoke, cigarette smoke (CS), and respiratory bacterial/viral infections can further lead to worsening of the existing exacerbation [10, 17, 21], which can lead to abnormal rhythmicity of lung function by altering the expression of circadian clock genes. However, it remains to be determined if alternative emerging tobacco products, such as WPS and e-cig vapor containing nicotine, similarly affect the expression of circadian clock proteins/genes in the mouse lungs. Our results indicate that acute exposure to WPS and e-cig vapor containing nicotine differentially alters the abundance and expression levels of circadian clock genes in mouse lung.

In the present study, we showed that an acute 10-day WPS exposure leads to changes in the expression levels of circadian clock genes. Recently, we have shown that cigarette smoke/environmental tobacco smoke can disconcert the molecular clock by disrupting the SIRT1 signaling pathway, leading to reduced expression of BMAL1 in both mice and patients with COPD [10, 12]. This study suggests that the molecular clock dysfunction can potentiate lung inflammatory responses to environmental stressors such as environmental tobacco smoke. Comparatively, another study showed that cigarette smoke induced transcriptomic alterations in pulmonary circadian clock gene expression, leading to CS-induced phase shift in the rhythmic expression of *Bmal1* [22]. We found that the expression of lung-specific core clock genes (*Clock*, *Bmal1*) as well as clock controlled output genes (*Rev-erbα*, *Per2*, *Rev-erb β*, *Cry-2*, *Rorα*) showed a differential pattern of expression in the lungs of mice exposed to acute WPS. More specifically, the expression of *Rev-erbα*, *Clock*, *Cry2*, *and Rorα* genes in lung tissues were upregulated in WPS exposed mice, suggesting that WPS exposure induces changes in pulmonary clock gene expression. A study in the murine model has shown that CS can affect the transcriptional regulation of *Rev-erbα* thereby disrupting lung circadian rhythmicity that may play an important role in CS-induced lung pathophysiology [13].

We and others have shown that acute exposure to WPS leads to increased oxidative stress, inflammatory cell influx, pro-inflammatory mediators release and lipid peroxidation within mouse lung [1, 23]. It has also been shown that the molecular clock is involved in redox regulation of lung inflammation, oxidative stress, DNA damage and cellular senescence [14, 20, 24–28]. It is possible that enhanced inflammatory responses in the lung following WPS exposure can disrupt circadian clock gene expression which can further potentiate lung inflammatory and oxidative stress responses. It has been shown that transcription of *Rev-erbα* regulates redox-mediated oxidative stress and inflammation through nuclear factor kappa B (NF- κB) in a neonatal hyperoxia mouse model [29]. We found a significant change in the expression of *Rev-erbα* following acute exposure to WPS suggesting that transcription of *Rev-erbα* is susceptible to inflammatory and oxidative stress responses imposed by WPS which can further aggravate cellular function and injury [30]. The molecular clock output proteins REV-ERBα and REV-ERBβ modulate and control hemostatic pulmonary inflammation and metabolism in epithelial cells and macrophages [11, 31–33]. The reason for the alteration of circadian clock proteins and genes by WPS is not known. It may be related to the level of carbon monoxide (CO) and particulates generated by WPS which alters the molecular clock in another system [34, 35]. The results of the present studies concur with results of previous studies on the effect of tobacco-based products [10–12, 17, 36], like conventional cigarettes, in exacerbating pulmonary circadian clock alternations through changes in expression of clock proteins.

We found that the exposure to e-cig vapor containing nicotine also affects the expression levels of circadian clock genes in mouse lungs, though the effect was milder compared to WPS exposed samples. A considerable body of evidence is available suggesting that nicotine can cause a phase shift in the circadian rhythm of rats mediated by nicotinic acetylcholine receptors (nAChRs) in the SCN [37]. Since, airway epithelium expresses this receptor abundantly nicotine can binds to the apical cell surface of the epithelium, where the expression of nAChRs is the highest [38], disrupting circadian rhythmicity in the lungs. It has been shown previously that the nicotine administration dose equivalent to that of smoking a single cigarette is enough to disrupt neuronal activity in the SCN [39]. It is possible that e-cig vapor containing nicotine contributes to the disruptions in the rhythmicity of circadian clock genes in other peripheral organs in addition to the lungs. However, it is more likely that inhaled nicotine-mediated transcriptional alterations of circadian clock genes can affect lung circadian rhythm differently than nicotine circulating in the body or administered via subcutaneously.

Complementing our findings, a recent study investigating the effects of e-liquid solvents on circadian clock dysfunction showed that propylene glycol and glycerol themselves elicit changes in clock gene expression within the lungs [40]. Although the focus was only on the propylene glycol and glycerol solvents of e-liquids, the results suggest that pulmonary circadian clock dysfunction is exacerbated by changes in the expression of core clock and clock-controlled output genes [40]. Though our data cannot confirm the biological significance of alteration of the circadian clock genes *per se* by PG/PG and nicotine, it does show that acute exposure to e-cig vapor could alter the expression of circadian clock genes that may have repercussions on the biological functions of the lung. Similarly, we have shown the differential disruption of the circadian molecular clock by conventional tobacco smoke in mouse and human lungs [10, 12]. Our present results also show a similar alteration, albeit to a different extent, on the molecular clock by two different tobacco products. We did not use data from our previous CS exposures to compare different tobacco products, but it is conceivable that similar pathophysiological changes may ensue exacerbating pulmonary cellular alterations via the molecular clock. Further, we did not study the effects of acute exposure to WPS or e-cig vapor on circadian molecular clock gene expression at different time points. It would have been more informative to look into the effects of WPS and e-cig vapor containing nicotine on the timing (peak phase) and amplitude of the daily rhythm of circadian clock genes in mice.

**Fig 7:**
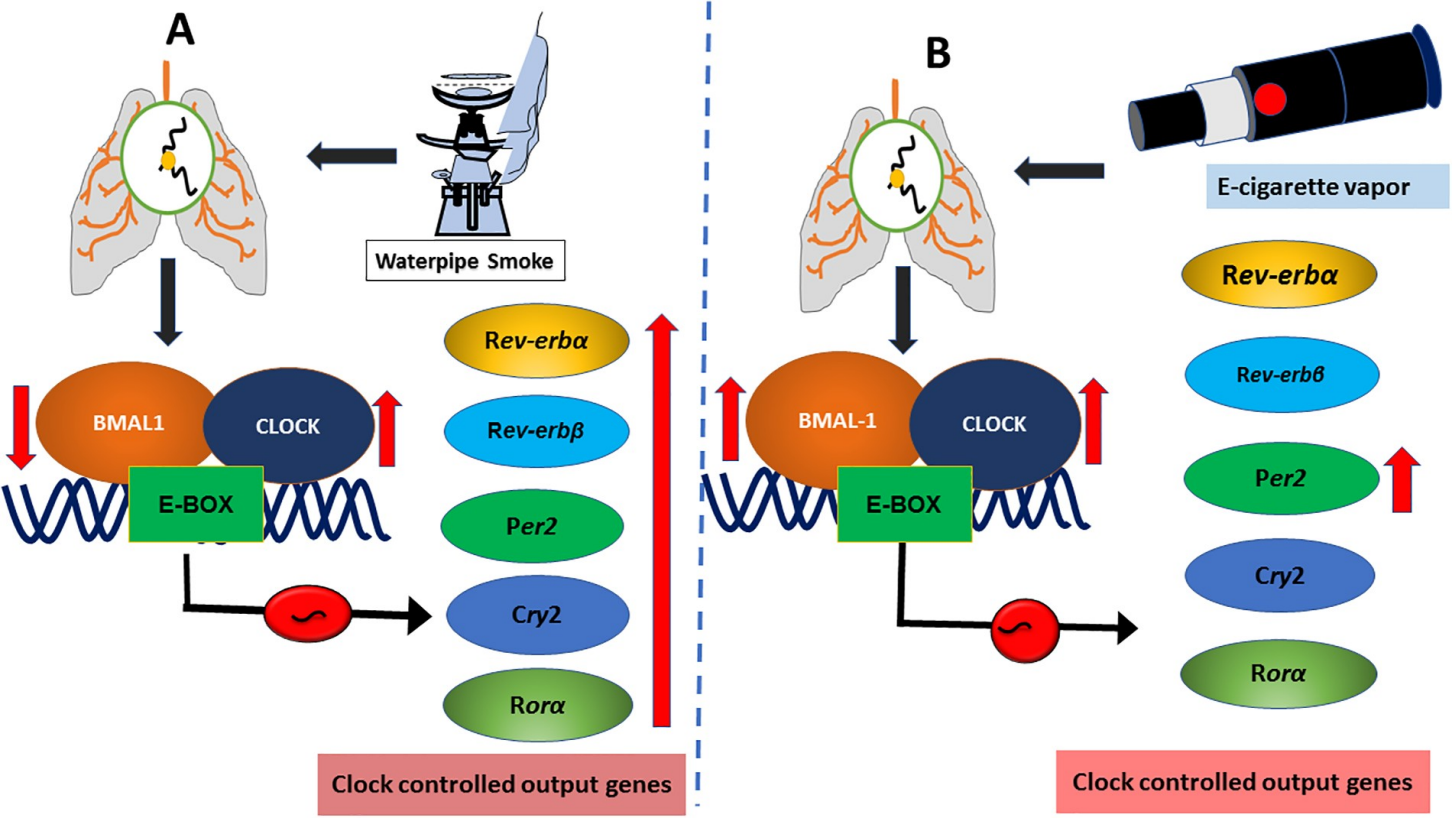
Schematic representation of waterpipe smoke (WPS) and e-cigarette vapor containing nicotine-induced alterations in the expression and abundance of circadian molecular clock genes. Exposure to alternative tobacco products, such as (A) WPS and (B) e-cig vapors containing propylene glycol with nicotine affects the expression of circadian molecular clock genes in mouse lungs. Molecular clock consists of a heterodimer of transcription factors BMAL1and CLOCK. The BMAL1:CLOCK heterodimer starts transcription of *Per2* and *Cry2* by binding to specific DNA elements (E-box) located in the promoter region. Altered expression of circadian clock genes may be a result of acute WPS and/or e-cig exposure induced oxidative stress and lung inflammatory response. This can negatively affect pulmonary function during chronic exposures and thus may contribute to the pathobiology of chronic airway diseases. BMAL1, brain and muscle aryl hydrocarbon receptor nuclear translocator-like 1; CLOCK, circadian locomotor output cycles protein kaput; nuclear receptor subfamily 1 group D member 1 (*Nr1d1 or Rev-erbα)*; nuclear receptor subfamily 1, group D, member 2 (*Nr1d2 or Rev-erbβ*); *Rorα*, retinoic acid receptor-related orphan receptor α; period circadian regulator 2 (*Per2*); Cryptochrome 2 (*Cry2*).

Overall, our results support the hypothesis that exposure to WPS and e-cig vapor containing nicotine alters the expression of circadian clock genes in mouse lungs. Furthermore, our data reveal that WPS and e-cig vapors containing nicotine can differentially impact the expression/abundance of circadian clock genes in mouse lungs (Fig 7). Based on these results it is reasonable to propose that these alterations caused by WPS and e-cig nicotine can have a substantial chronic effect on clock-dependent lung pathophysiology. Moreover, we cannot conclude if the modulation of circadian molecular clock genes/proteins is of any biological relevance in acute exposure model of these emerging tobacco products on biological functions, e.g., mucus secretion, inflammation, DNA damage, or cellular functions. However, it is clear from our findings that WPS and e-cig vapors can modulate the circadian molecular clock genes differently which can affect the circadian molecular clock and could amplify, accelerate, or change the course of pathological processes during chronic exposure. Further studies are required to establish the role WPS and e-cig vapor have at different time points on relevant transcriptional regulators that may lead to circadian clock dysfunction to get a better insight of the differential effects of e-cig vapor and WPS on cellular and molecular functions of the lungs.

## Supporting information

S1 Fig Full gels/blots with bands showing abundance of circadian clock proteins in acute waterpipe exposed mouse lungs. (PDF)

S2 Fig Full gels/blots with bands showing abundance of circadian clock proteins in acute e-cigarette exposed mouse lungs. (PDF)
