# Supplementary material for: Waterpipe smoke and e-cigarette vapor differentially affect circadian molecular clock gene expression in mouse lungs
Source: PLoS One. 2019 Feb 27;14(2):e0211645. doi: 10.1371/journal.pone.0211645 (PMC6392409; doi:10.1371/journal.pone.0211645)

Supplementary File Information

Waterpipe smoke and e-cigarette vapor containing nicotine differentially affects the circadian clock gene expression in mouse lungs

Naushad Ahmad Khan*, PhD, Shaiesh Yogeswaran*, Qixin Wang*, PhD, Thivanka Muthumalage, PhD, Isaac K. Sundar, PhD, #Irfan Rahman, PhD

*These authors contributed equally

Full unedited gel for
Fig. 1(B) BMAL1

Gel-1 (10% Gel): BMAL1

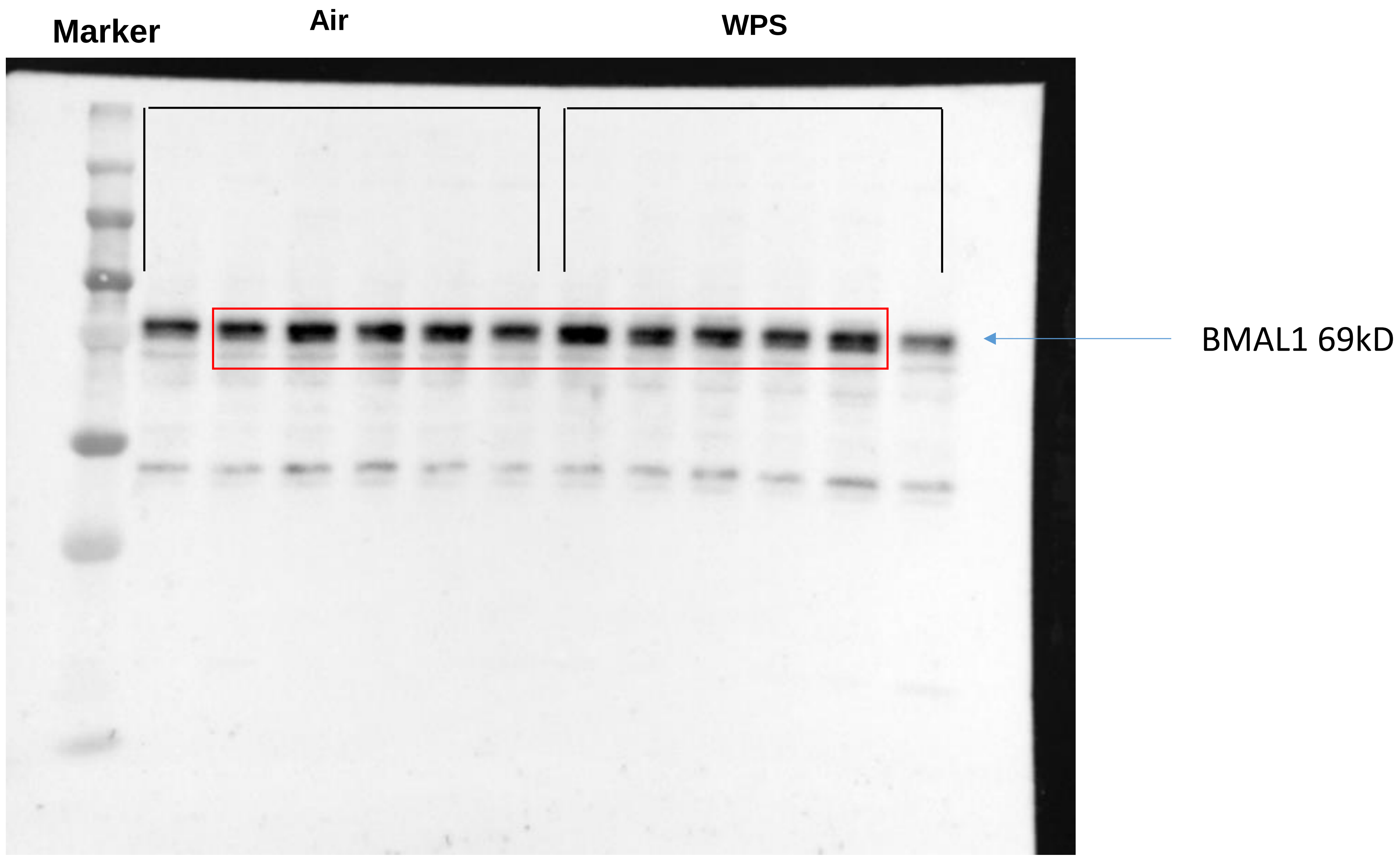

Full unedited gel for Fig. 1(C) Clock

Gel-1 (10% Gel): Clock

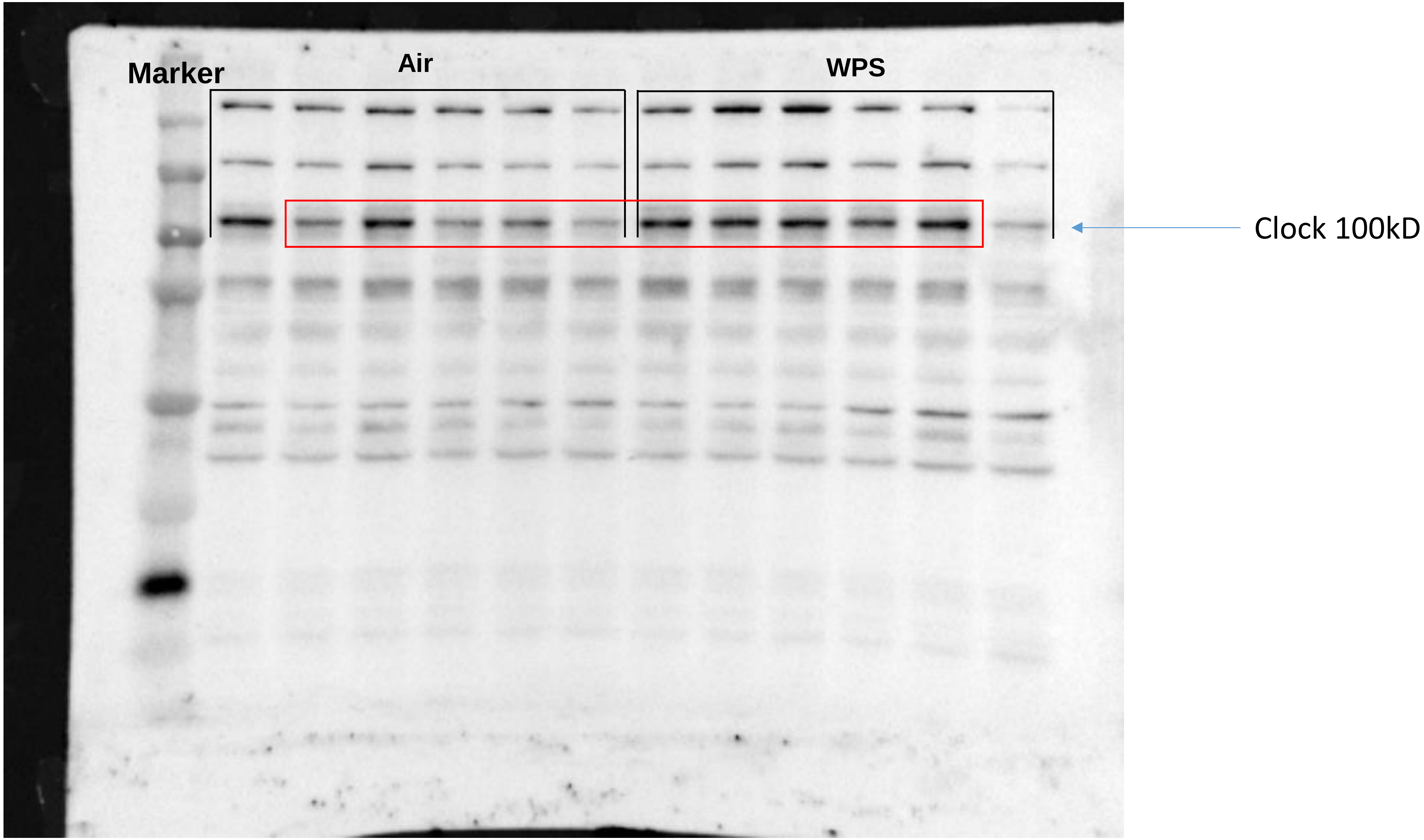

Full unedited gel for
Fig. 1(B,C) Beta-Actin

Gel-1 (10% Gel): Clock : β-Actin

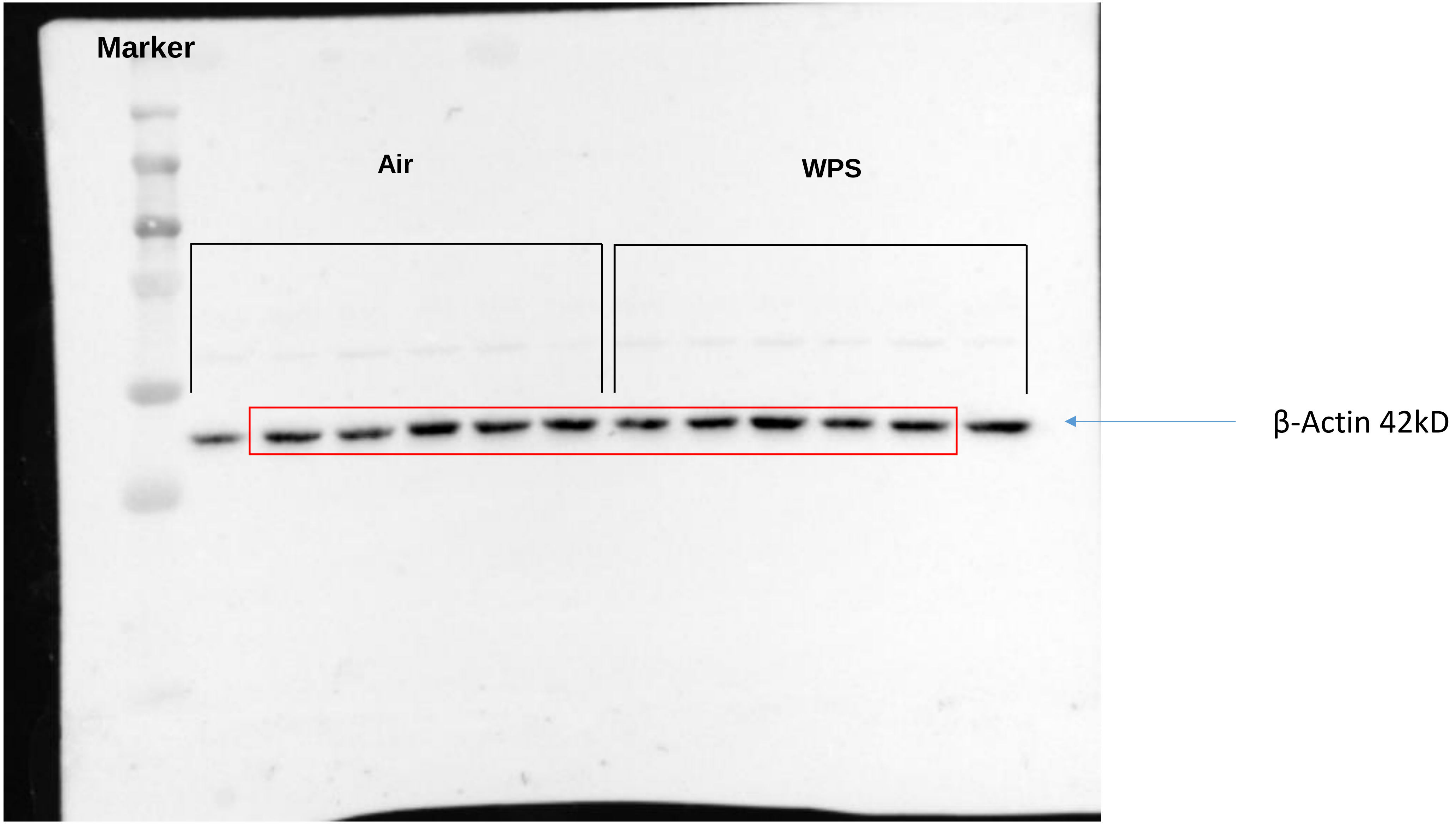

Full unedited gel for
Fig. 1(D) Per2

Gel-4 (10% Gel): Per2

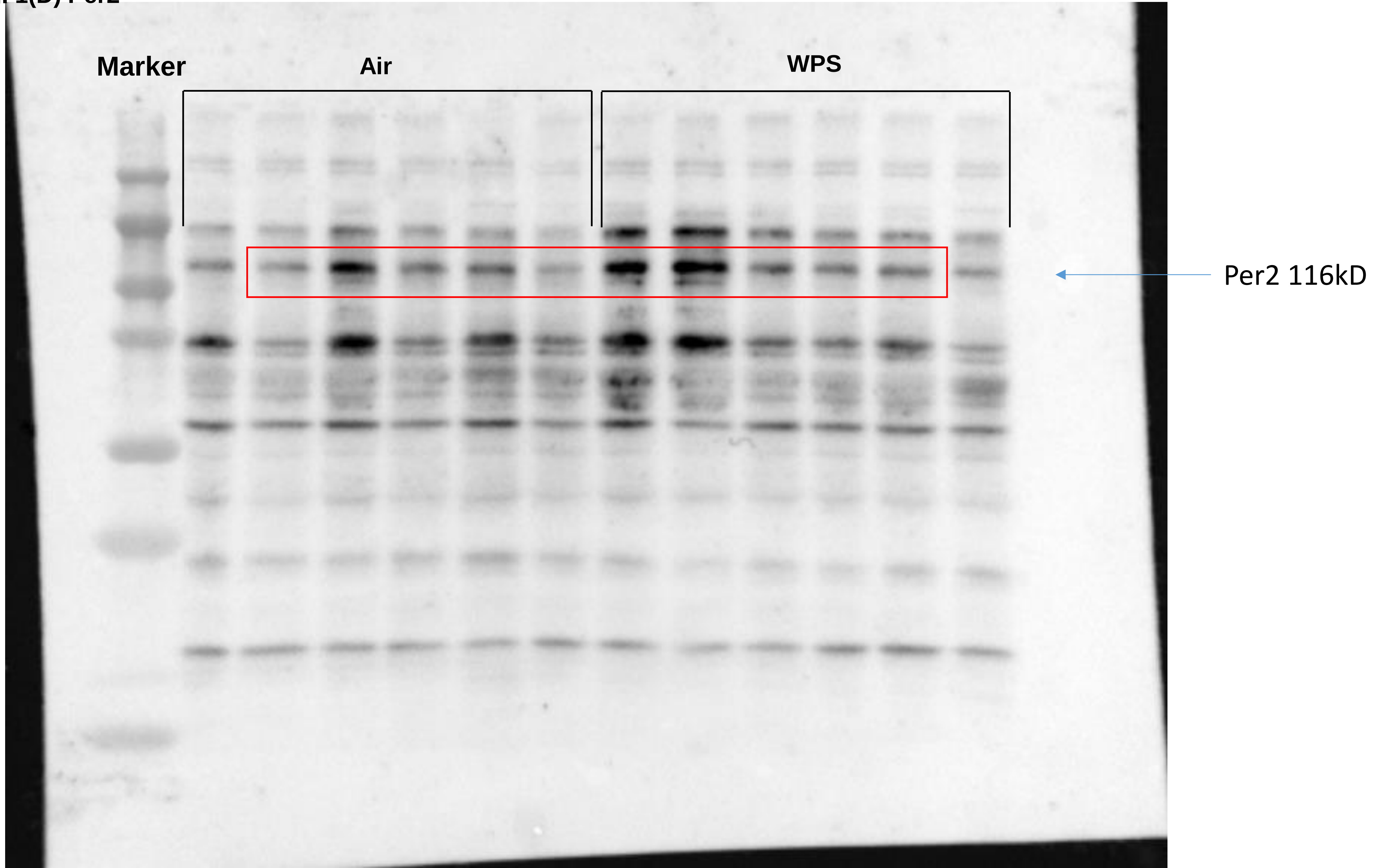

Full unedited gel for
Fig. 1(D) β-Actin

Gel-4 (10% Gel): Per2: β-Actin)

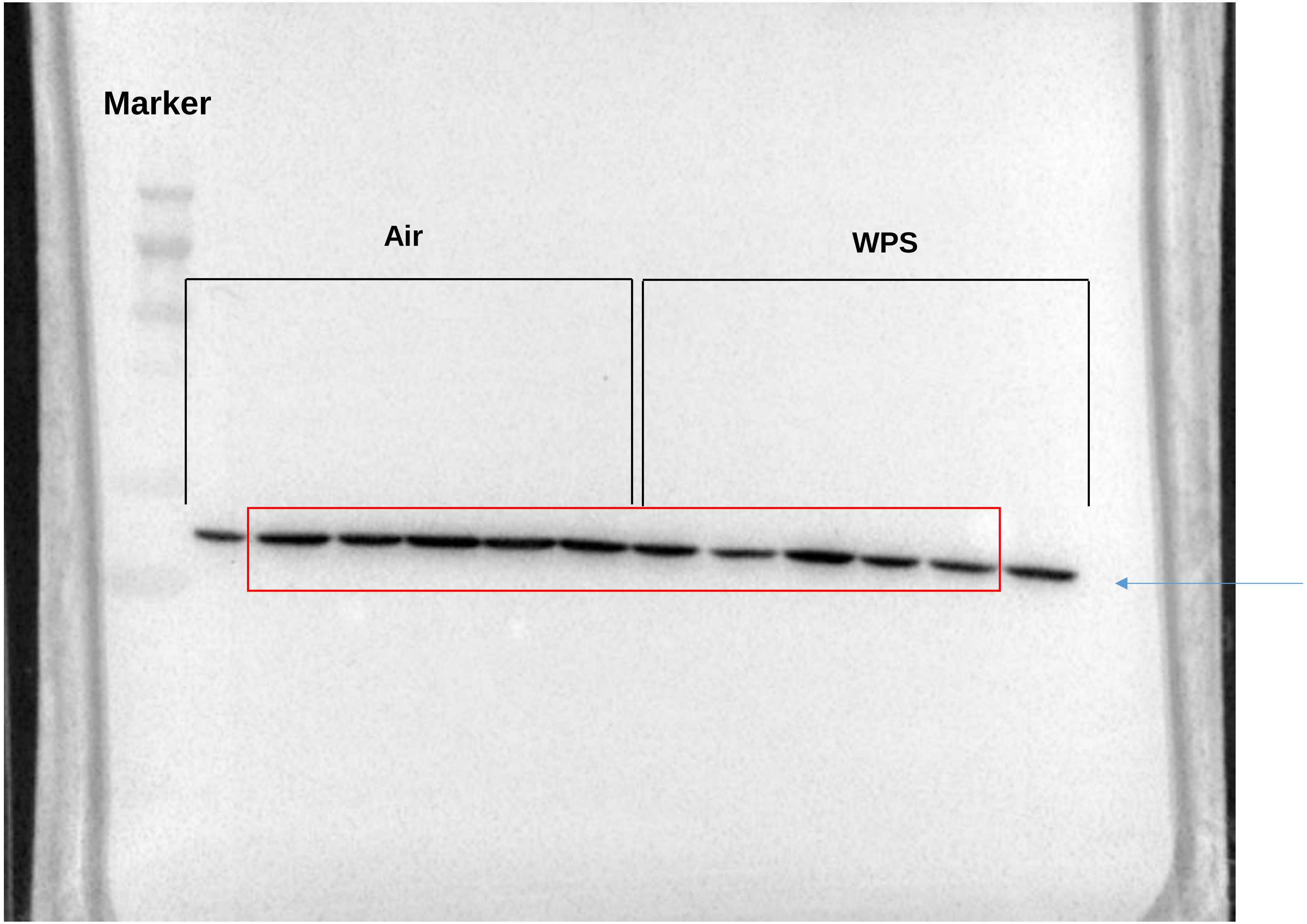

Beta-Actin 42kD

Full unedited gel for
Fig. 1(E) Rev-Erb Alpha

Gel-3 (10% Gel): Rev-Erb Alpha

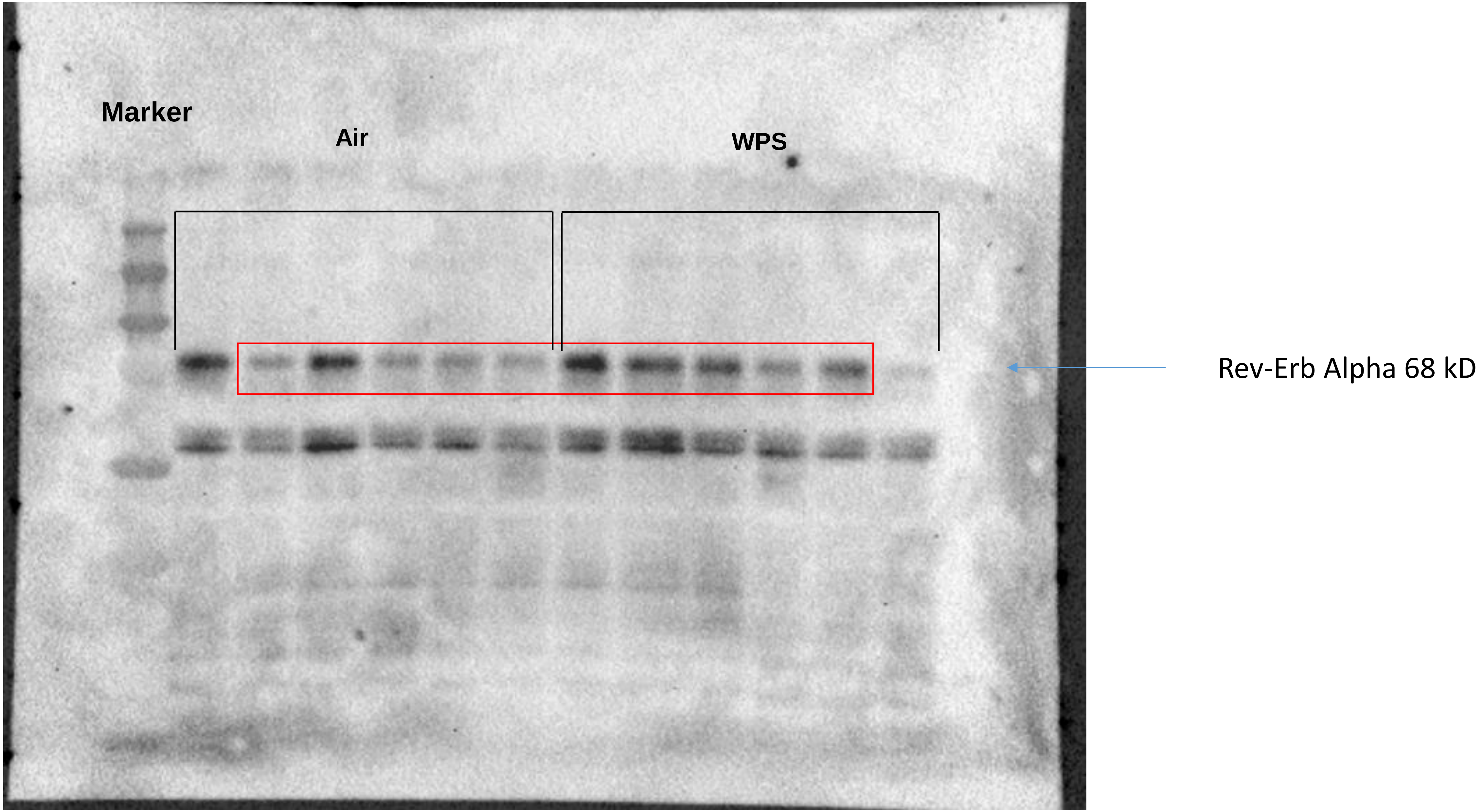

Full unedited gel for
Fig. 1(E) β-Actin

Gel-3 (10% Gel): Rev-Erb Alpha : β-Actin

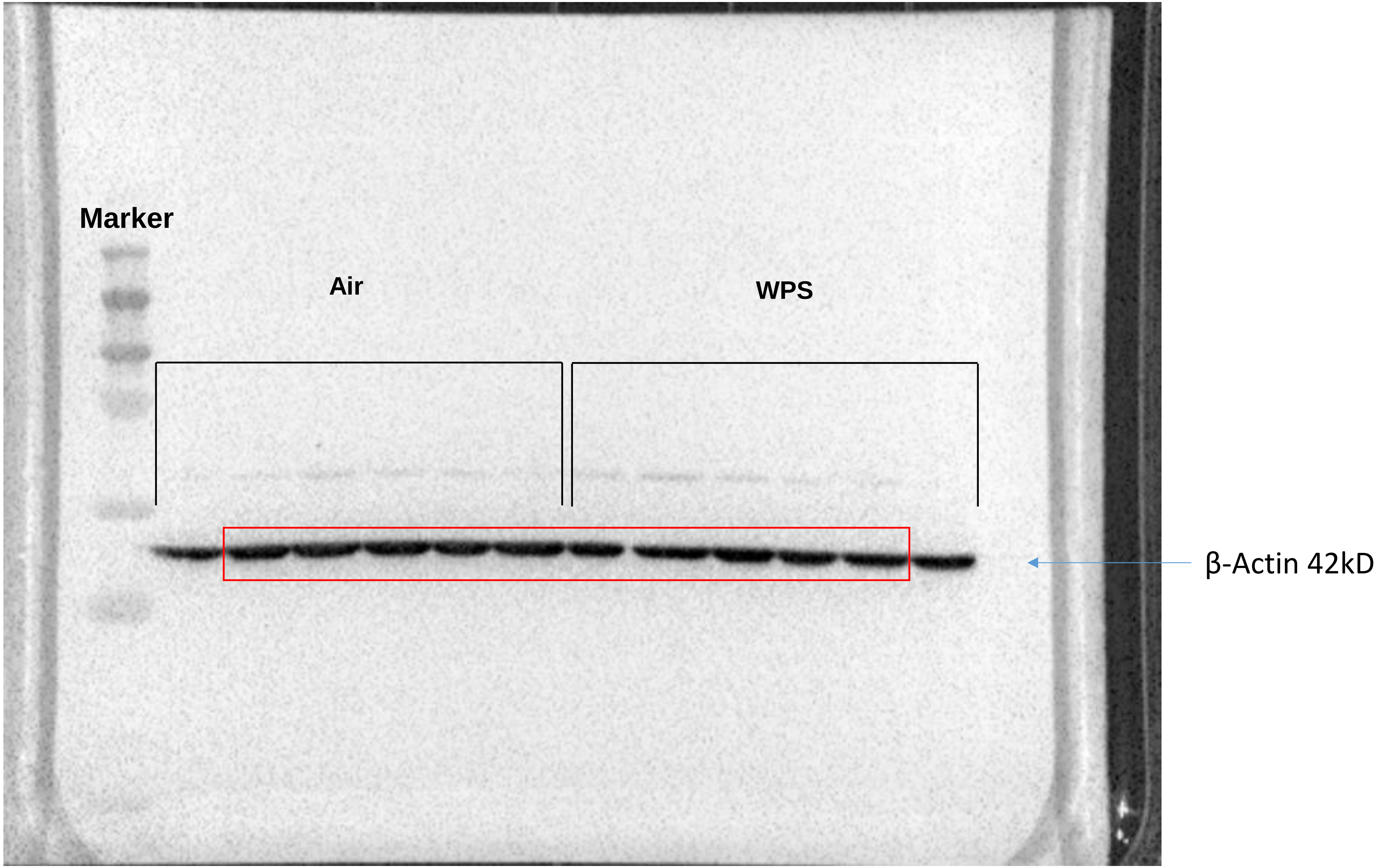

Supplement: S1 Fig — (PDF) [file pone.0211645.s001.pdf]
