# Supplementary material for: Waterpipe smoke and e-cigarette vapor differentially affect circadian molecular clock gene expression in mouse lungs
Source: PLoS One. 2019 Feb 27;14(2):e0211645. doi: 10.1371/journal.pone.0211645 (PMC6392409; doi:10.1371/journal.pone.0211645)

Gel-6 (10% Gel): Air, PG, and PG+Nicotine Exposed (PGN)   BMAL1

Full unedited gel for Fig. 2(B) BMAL1

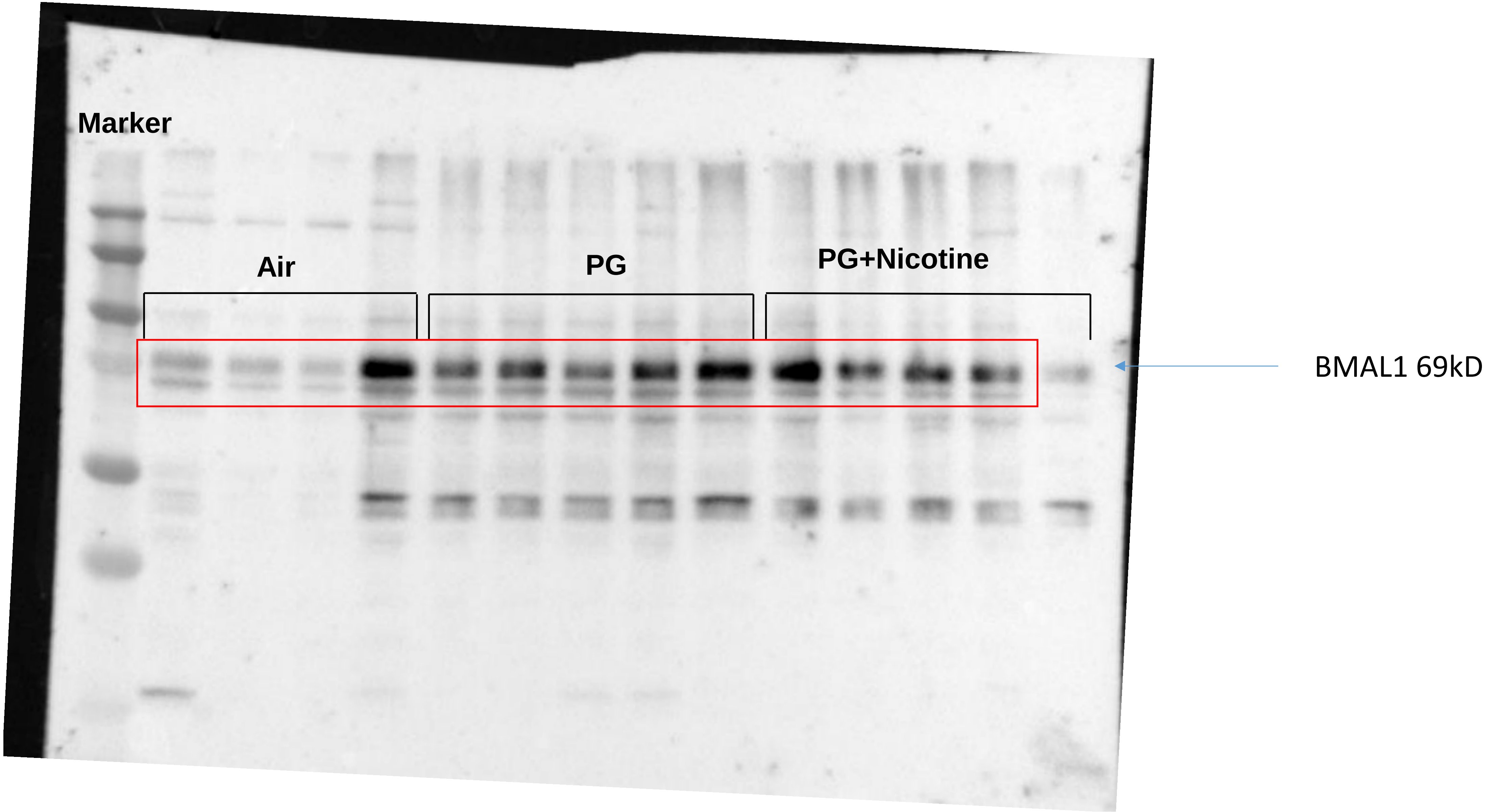

Gel-6 (10% Gel) : Air, PG, and PG+Nicotine Exposed (PGN)   BMAL1: β-Actin

Full unedited gel for Fig. 2(B) β-Actin

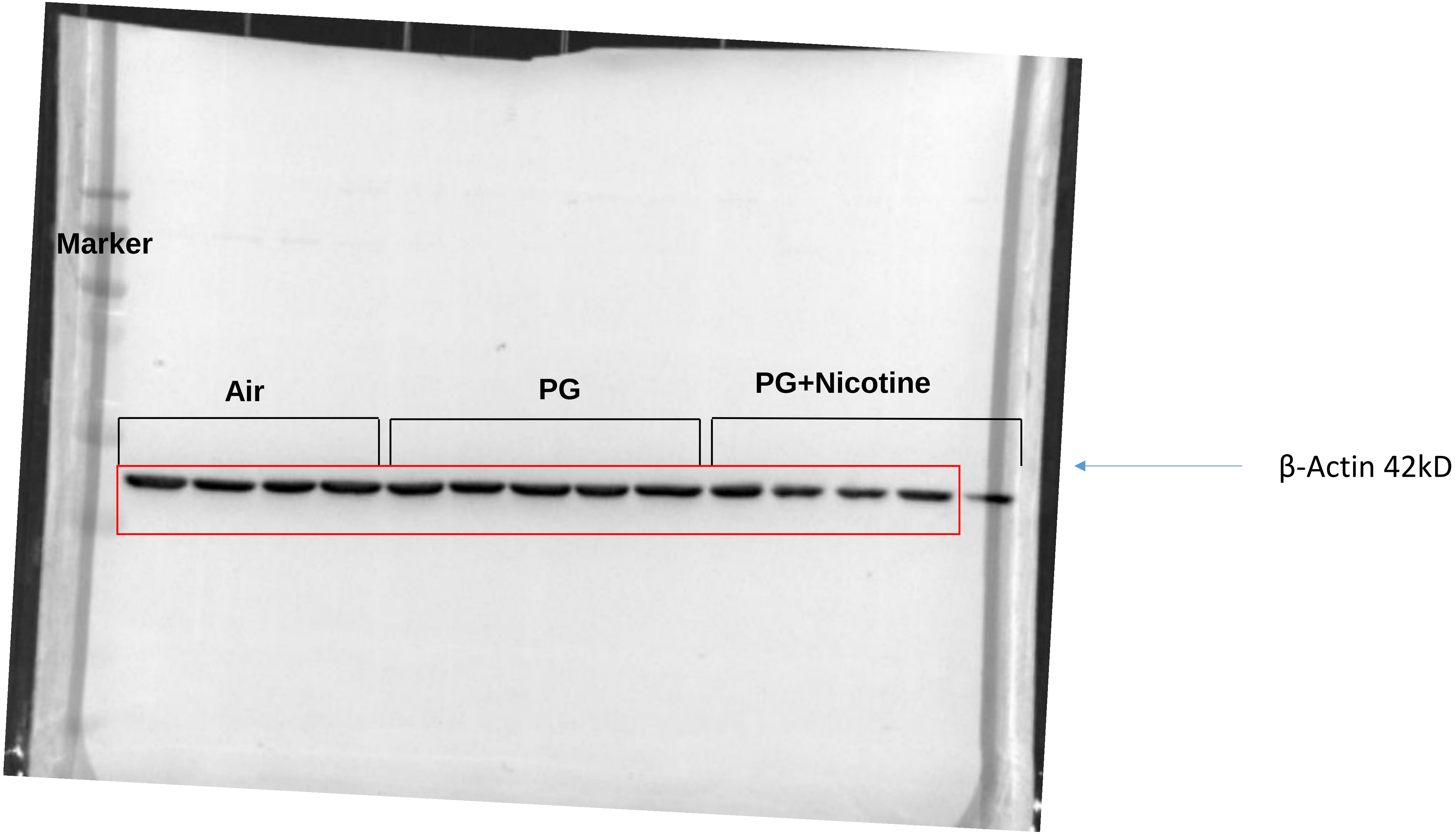

Gel-7 (10% Gel): Air, PG, and PG+Nicotine Exposed (PGN) Clock

Full unedited gel for Fig. 2(C) Clock

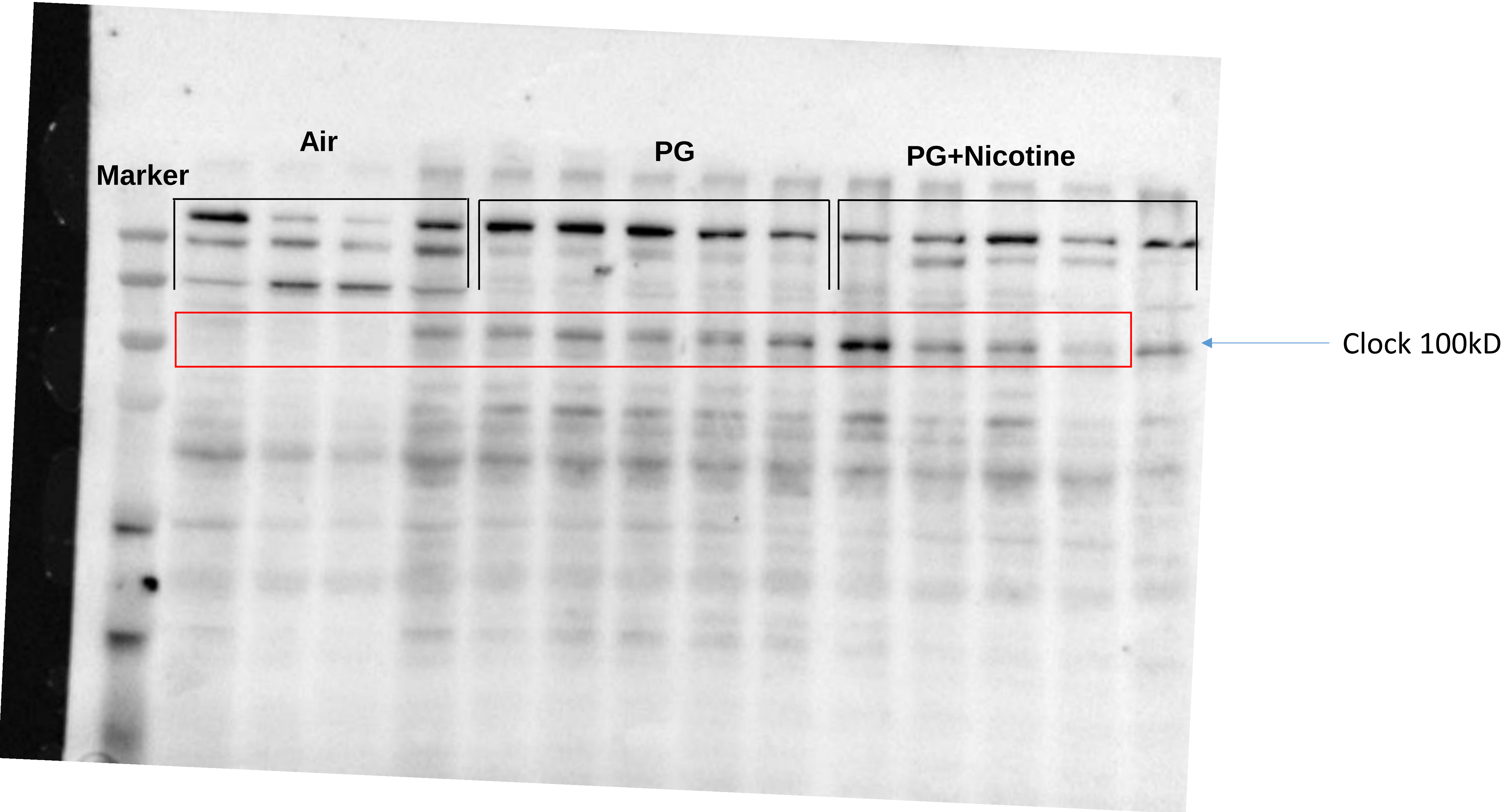

Gel-7 (10% Gel) : Air, PG, and PG+Nicotine Exposed (PGN)   Clock: β-Actin

Full unedited gel for Fig. 2(C) β-Actin

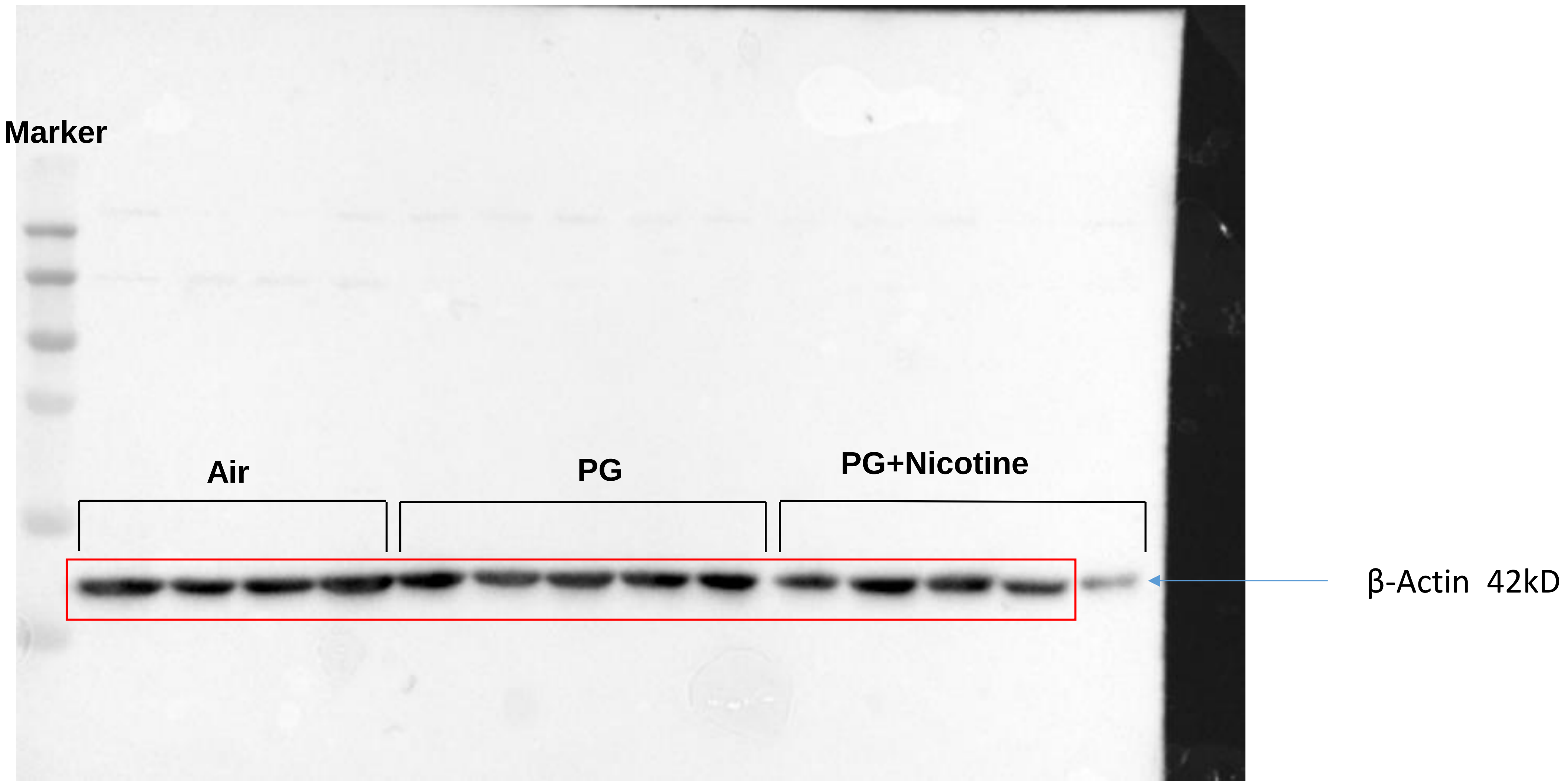

Gel-6 (10% Gel): Air, PG, and PG + Nicotine Exposed (PGN) Per2

Full unedited gel for Fig. 2(D) Per2

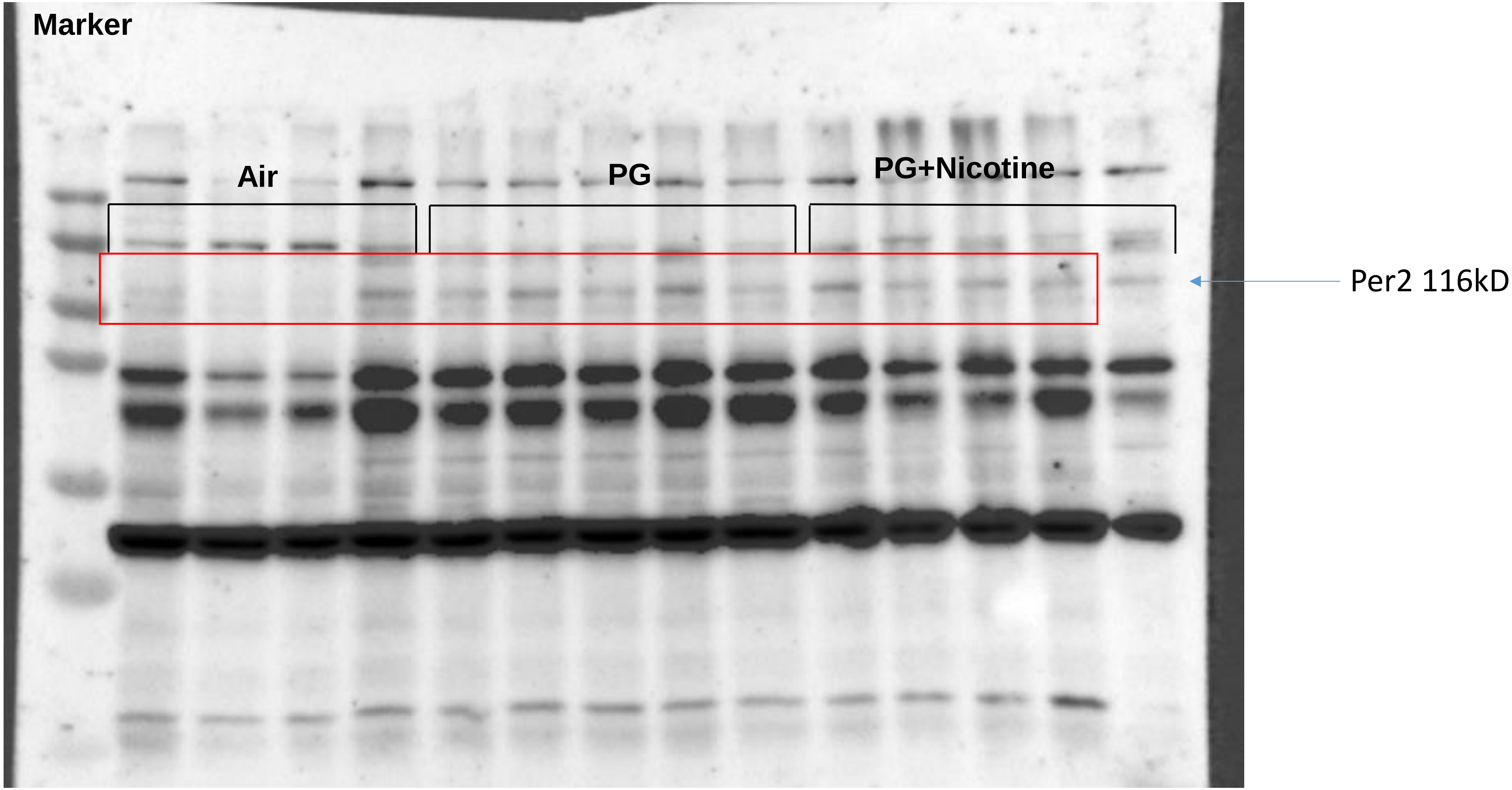

Gel-6 (10% Gel) : Air, PG, and PG + Nicotine Exposed (PGN)   Per2: β-Actin

Full unedited gel for Fig. 2(D) β-Actin

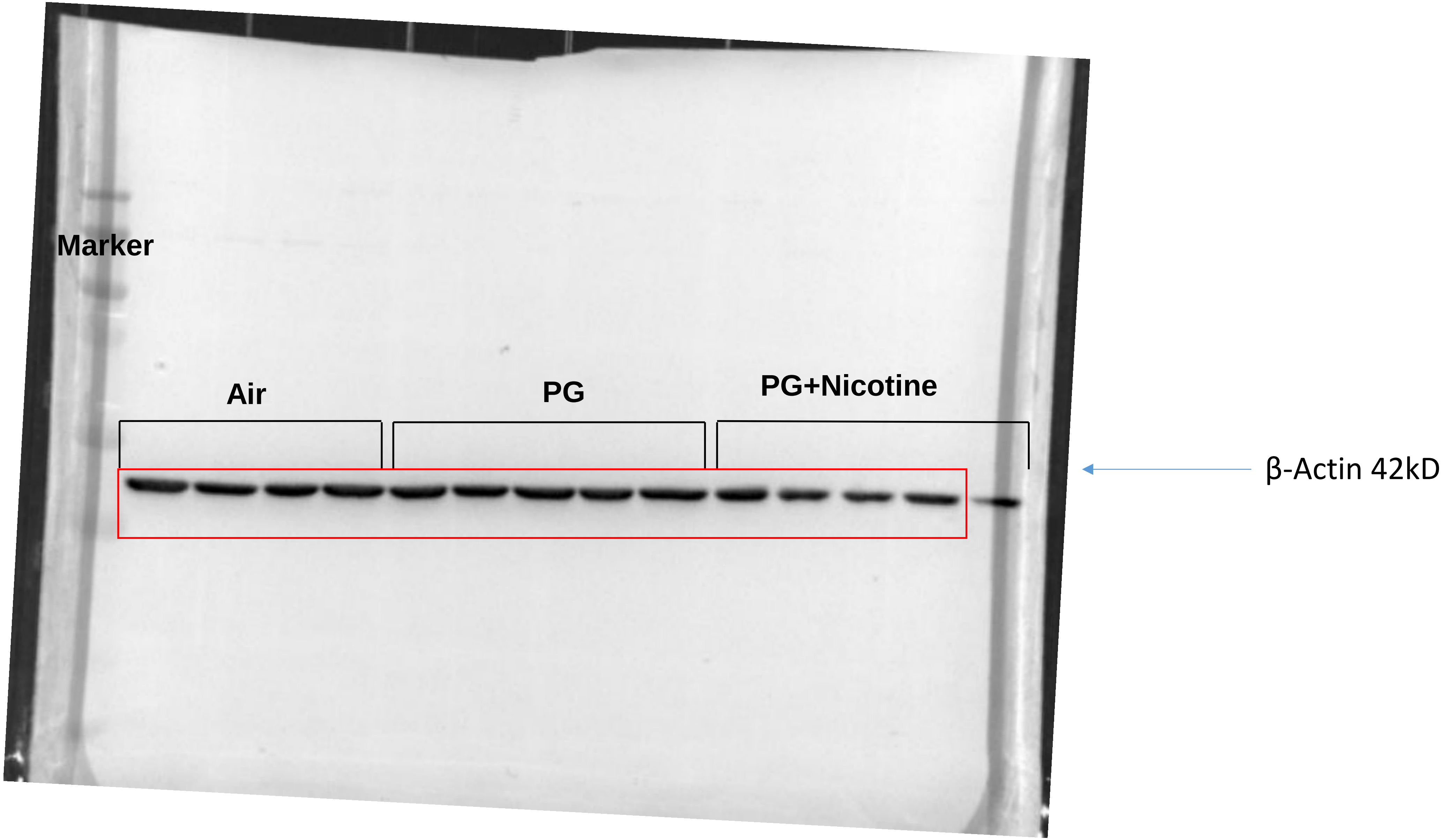

Gel-7 (10% Gel): Air, PG, and PG + Nicotine Exposed (PGN)   Rev-Erb Alpha)

Full unedited gel for Fig. 2(E) Rev-Erb Alpha

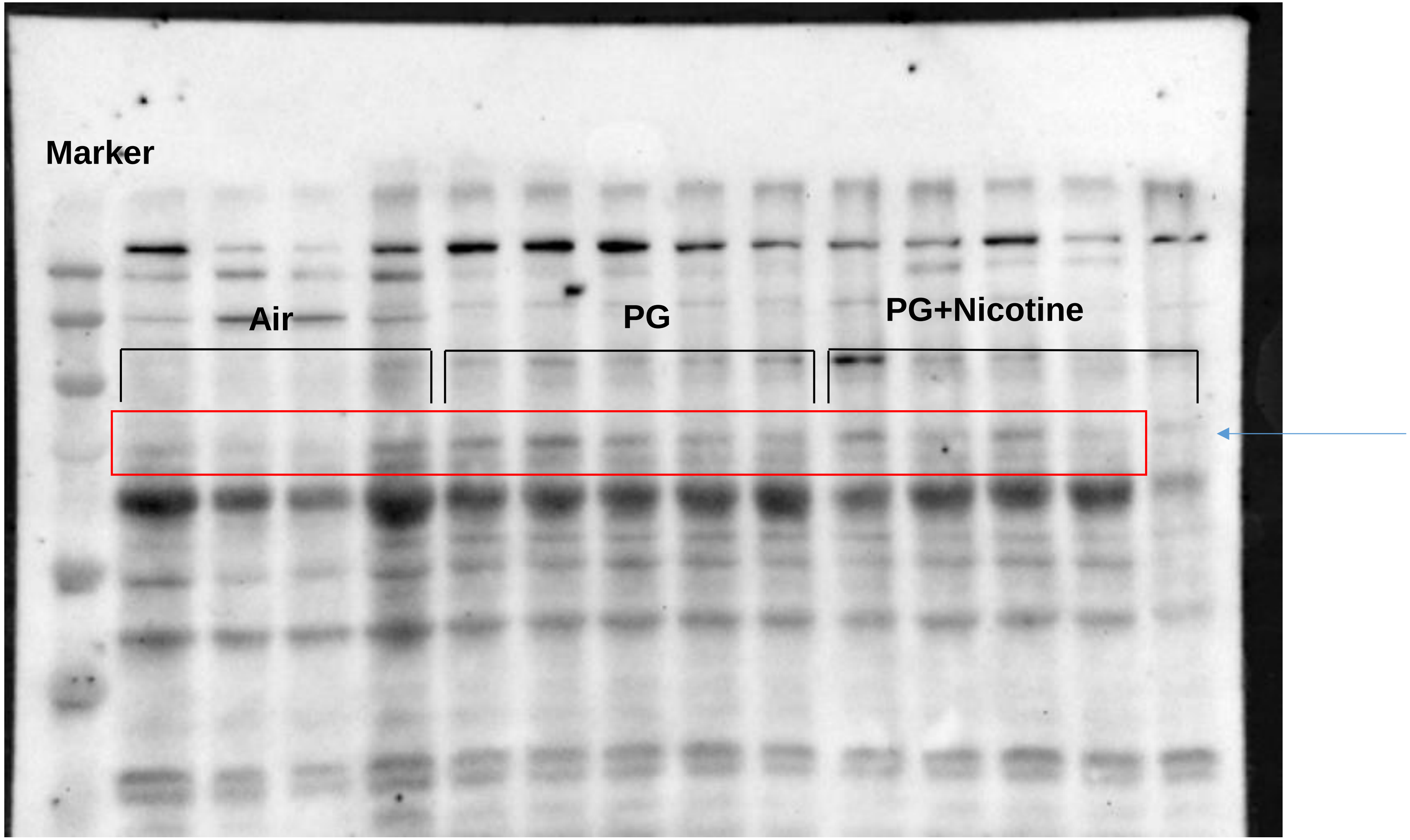

Rev-Erb alpha 68kD

Gel-7 (10% Gel): Air, PG, and PG+Nicotine Exposed (PGN) Rev-Erb Alpha: β-Actin

Full unedited gel for Fig. 2(E) β-Actin

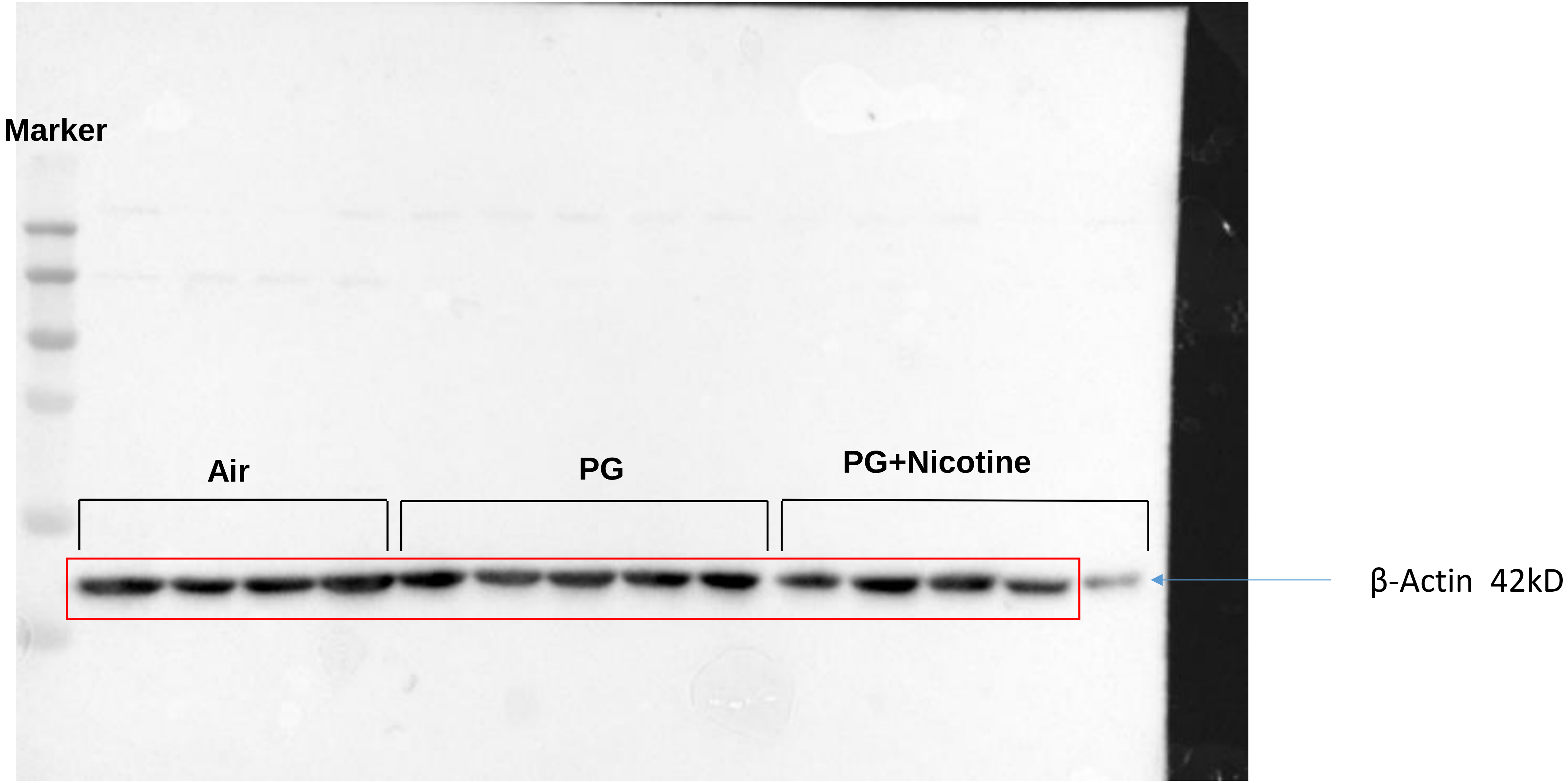

Supplement: S2 Fig — (PDF) [file pone.0211645.s002.pdf]
